# Progressively altered genes in colorectal carcinogenesis link oncogenesis immune cycle and tumor microenvironment

**DOI:** 10.1038/s41598-025-21401-y

**Published:** 2025-10-27

**Authors:** Bingwen Zhou, Qingrui Liu, Chuyue Huang, Hao Chen, Pei Wang, Yueyang Lu, Shujun Jiang, Desong Kong, Lu Wang, Zhimin Fan

**Affiliations:** 1 Jiangsu Clinical Innovation Center for Anorectal diseases of T.C.M, Nanjing Hospital of Chinese Medicine, Nanjing University of Chinese Medicine, Nanjing, China; 2 Anorectal center, Nanjing Hospital of Chinese Medicine affiliated to Nanjing University of Chinese Medicine, Nanjing, China; 3 Nanjing Hospital of Chinese Medicine, Nanjing University of Chinese Medicine, Nanjing, China; 4 Big data center, Nanjing Hospital of Chinese Medicine, , Nanjing University of Chinese Medicine, Nanjing, China

**Keywords:** Colorectal cancer, Tumor microenvironment, Normal-Adenoma-Cancer, Tumor immunology, Transcriptomics, Biomarkers, Diagnostic markers, Cancer, Cancer screening, Diseases, Cancer, Cancer microenvironment

## Abstract

The adenoma-adenocarcinoma pathway represents a crucial mechanism underlying the development of colorectal precancerous lesions, encompassing approximately 85%-90% of colorectal cancer (CRC). Elucidating the molecular mechanisms underlying colorectal cancer progression is of paramount importance for achieving early and accurate diagnosis as well as effective treatment. We collected peripheral blood mononuclear cells (PBMC) from healthy controls, adenoma patients, and adenocarcinoma patients, and performed transcriptomic profiling to characterize dynamic gene expression during carcinogenesis. Diagnostic potential was assessed using receiver operating characteristic (ROC) analysis, and a random-forest model was trained to classify disease status. The screening identified genes with consistent expression changes as potential early diagnostic markers, and further exploration of their functions and significance in CRC is conducted through analysis of the TCGA database. The findings revealed that the progression of precancerous lesions in the “Normal-Adenoma-Cancer” (N-A-C) sequence was accompanied by a sustained enhancement of the immune response. Notably, HECW2, WARS1, SLC16A3, SECTM1, IFITM3, ADAMTSL4, FCGR1A, F2RL1, OPLAH, SERPINA1, FCGR1CP showed consistent upregulation with promising diagnostic performance. In our PBMC cohort, the random-forest classifier achieved an accuracy of 93.62%, indicating potential for distinguishing cancer from precancerous lesions. The bioinformatics analysis revealed a significant association of these genes with DNA methyltransferase, DNA mismatch repair, m6A regulator, tumor mutational burden (TMB) and microsatellite instability (MSI). Furthermore, a detailed analysis was further performed on WARS1. In the “N-A-C” sequence, WARS1 exhibited a significant upregulation in both blood and tissues, demonstrating a positive correlation with augmented infiltration of immune cells, activation of stromal and immune responses, as well as heightened activity during the cancer immune cycle. However, it demonstrates a declining trend in the progression of CRC from stage I to IV, which may be intricately associated with the metastasis of CRC. The WARS1 can serve as a reliable indicator of the immune response in CRC, thereby demonstrating its potential to impede tumorigenesis or metastasis.

**Supplementary Information:**

The online version contains supplementary material available at 10.1038/s41598-025-21401-y.

## Introduction

Colorectal Cancer (CRC) is one of the most prevalent malignant neoplasms affecting the gastrointestinal tract. CRC ranks as the third most prevalent malignancy worldwide, accounting for over 1.85 million cases and causing approximately 850,000 deaths annually^1^. Recently, there has been a rising incidence of CRC in younger individuals and it is projected that the burden of this disease will increase by 60% by 2030^2^. CRC is a complex disease influenced by multiple factors, including genetics, environment and lifestyle^3^. CRC is a malignant tumor that originates from the mucosal epithelium of the colon and rectum. Recent studies have identified three primary pathogenic mechanisms, including the “Normal-Adenoma-Adenocarcinoma” (N-A-C) pathway, the “inflamma-cancer” transformation pathway and the “De-Novo” pathway. These pathways are characterized by genetic mutations and chromosomal instability^4,5^.

Inactivation of the APC tumor suppressor gene represents the initial event in CRC development, initiating the formation of aberrant crypt foci and facilitating its progression towards adenoma. The majority of adenomas progress to CRC through the accumulation of activating mutations in Kras followed by sequential mutations in Wnt, EGFR, P53, SMAD4 and TGF-β signaling pathways^6,7^. Additionally, a subset of CRC arises through distinct molecular pathways, such as the activation of BRAF mutations or the inactivation of DNA mismatch repair genes^8,9^.

From a clinical perspective, adenoma serves as a prototypical precancerous lesion that can be effectively excised during colonoscopy, while colectomy is considered for cases where direct removal proves challenging or entails significant surgical risk. The risk of adenoma carcinogenesis is primarily determined by the patient’s medical history, lifestyle and the pathological characteristics of the adenoma. For instance, advanced age, a family history of cancer, tobacco smoking, absence of non-steroidal anti-inflammatory drug usage and presence of large adenomas (diameter ≥ 1 cm) with tubulovillous or villous histology and high-grade dysplasia significantly elevate the risk of adenoma malignancy^5,10,11^. Is it feasible to evaluate the risk of developing adenomas or adenoma carcinogenesis at an earlier stage than the aforementioned assessment system? Novel biomarkers or innovative assessment systems are imperative for advancing the field.

The N-A-C sequence undergoes a continuous process of change, initiated by the APC gene and connected through sequential mutations. It is plausible that certain pathways or genes exhibit ongoing alterations in conjunction with the progression of this sequence. Based on the aforementioned hypothesis, this study commences with the N-A-C sequence and screens for key genes that follow its progression. Furthermore, we investigate the correlation between these key genes and CRC diagnosis, prognosis, tumor microenvironment as well as drug gene characteristics. It is anticipated that this study will enhance our comprehension of the pathogenesis of N-A-C and identify novel targets for early clinical diagnosis and prevention of colorectal carcinogenesis. Among them, WARS1 (tryptophanyl-tRNA synthetase, TrpRS) is classically known for its canonical role in protein synthesis through aminoacylation^12^. Beyond this, accumulating evidence has revealed non-canonical functions of WARS1 in immune regulation and angiogenesis, and dysregulated expression has been reported in several cancers, including colorectal cancer^13^. However, its specific role in CRC development remains poorly defined. In particular, no study has systematically investigated its expression dynamics across the N-A-C sequence, nor evaluated its potential diagnostic value in peripheral blood. In this work, we integrate peripheral blood mononuclear cell (PBMC) transcriptomes, tissue datasets, immunohistochemistry, and large-scale databases (TCGA) to assess WARS1 expression and its associations with mutational landscape, including tumor mutational burden (TMB), microsatellite instability (MSI), and copy number variation (CNV), as well as signaling pathways and immune–stromal interactions. This approach provides the first comprehensive characterization of WARS1 in the context of the N-A-C sequence, aiming to enhance understanding of CRC pathogenesis and identify novel targets for early diagnosis and prevention.

## Materials and methods

### Collection and preparation of PBMC

Volunteers were recruited from normal (*n* = 11), adenoma (*n* = 12) and colorectal cancer (*n* = 12) patients to collect blood samples (no prior chemotherapy, radiotherapy, or medication). Potential confounders such as comorbidities and lifestyle were not controlled. Informed consent was obtained from all participants or their legal guardians. PBMC were prepared as described previously^14^. After centrifugation (10 min, 25℃, 3000 rpm/min), the blood samples were divided into plasma layer and blood cell layer. The interface of the two layers was aspirated and added to a 15mL centrifuge tube containing 4mL of RPMI-1640 medium, the medium was adjusted to 8mL to make the cell suspension. The cell suspension was added slowly to a centrifuge tube containing 4mL Ficoll lymphocyte separation solution and then centrifuged (25 min, 25℃, 1500 rpm/min). After centrifugation, the suspension was divided into 4 layers. PBMC was aspirated and added to RPMI-1640 medium, the liquid was supplemented with medium to 10-12mL, mixed and centrifuged (10 min, 25℃, 1500 rpm/min). after the supernatant was discarded, the medium was added to 10mL and centrifuged (10 min, 25℃, 1500 rpm/min). PBMC was collected for subsequent analysis after removal of the supernatant.

### Transcriptome analysis of PBMC

Total RNA integrity was assessed using the RNA Nano 6000 Assay Kit on the Bioanalyzer 2100 system (Agilent Technologies, CA, USA). For each library, no less than 1 µg of total RNA (RIN ≥ 7) was used as input material. Poly(A) + mRNA was purified from total RNA using oligo(dT)-attached magnetic beads and then fragmented under elevated temperature. First- and second-strand cDNA synthesis was performed, followed by end repair, adaptor ligation, and PCR amplification. The resulting cDNA libraries (insert size ~ 370–420 bp) were purified using the AMPure XP system (Beckman Coulter, USA), and library quality was confirmed on the Agilent Bioanalyzer 2100 system. RNA sequencing was conducted on the Illumina platform. Each sample yielded 43.7 million clean reads (range: 39.8–48.5 million), corresponding to 6.6 Gb clean bases per sample. Sequencing quality was high, with an error rate ~ 0.03%, Q20 ~ 97%, Q30 ~ 92%, and GC content ~ 50%.

### Screening of differential genes with persistent changes

Differential expression analysis of two groups (Normal vs. Adenoma, Adenoma vs. Cancer) was performed using the DESeq2 R package. Normalization of raw counts was performed using the median-of-ratios method implemented in DESeq2. DESeq2 provides statistical routines for determining differential expression in digital gene expression data using a model based on the negative binomial distribution. The resulting P-values were adjusted using the Benjamini and Hochberg’s approach for controlling the false discovery rate. Gene with adjusted P-value ≤ 0.05 and |Log2FC| > 1 found by DESeq2 were assigned as differentially expressed for subsequent analysis.

### Cancer association analysis

For TCGA datasets, TPM values were used in subsequent analyses. The expression profiling of DNA mismatch repair genes (EPCAM, PMS2, MSH2, MSH6, MLH1), m^6^A regulators (YTHDF1-3, YTHDC1-2, LRPPRC, IGF2BP1-3, HNRNPC, HNRNPA2B1, FMR1, ELAVL1, FTO, ALKBH5, ZC3H13, WTAP, RBM15/15B, METTL3/14, VIRMA, CBLL1) and DNA methyltransferases (DNMT1/2/3A/3B) of each sample was extracted from COAD and READ datasets. The TMB and MSI data were also obtained from the TCGA project. The spearman correlation analysis was performed to investigate the association of progressively altered genes with the colorectal cancer.

### Estimation of immunological features

By applying gene set variation analysis (GSVA) package, a computational approach for inferring leukocyte representation in bulk tumor transcriptomes, the abundance of immune cells infiltrating the tumor was estimated^15^. A total of 122 immunomodulatory factors, including MHCs, immune receptors, immune chemokines and immune stimulators were compiled from Charoentong et al.^16^. Moreover, the investigation conducted by Auslander et al. yielded valuable reference into immune checkpoint molecules^17,18^. The infiltration of stromal and immune cells in CRC tissues was assessed using the ESTIMATE algorithm, which estimates the abundance of these cell types based on mRNA expression data from malignant tumors^19^. The cancer immunity cycle was curated based on previous research and the activities of all steps were estimated using single-sample gene set enrichment analysis (ssGSEA) derived from GSVA^20–22^.

### Collection of CRC datasets

In addition to the transcriptome data, we also downloaded COAD and READ data from TCGA (https://portal.gdc.cancer.gov/) for subsequent computational. The GSE20916 and GSE117606 datasets were collected from the Gene Expression Omnibus (GEO; https://www.ncbi.nlm.nih.gov/geo/) project^23,24^. The somatic mutational data were visualized using the maftools package, as implemented in R^25^. The GISTIC2.0 algorithm was employed for the analysis of amplification and deletion events based on copy number variation (CNV) data^26^.

### Construction of gene sets of known biological process

Gene sets encompassing cell cycle regulators, WNT pathway, mismatch repair, homologous recombination, nucleotide excision repair, DNA replication, cell cycle, Fanconi anemia, KEGG discovered histones, angiogenesis, FGFR3-relevant gene signatures, epithelial–mesenchymal transition 1–3 (EMT1-3), immune checkpoints, antigen processing machinery, pan-fibroblast TGF-β response signature (pan-F-TBRS), DNA damage repair, as well as CD8 + T effector were curated from previous literature^27,28,29^. the quantification of biological process activities was performed using the ssGSEA algorithm.

### Functional enrichment analysis

The GSEA analysis was performed to investigate the differential activation of signaling pathways in two distinct subgroups, using the gene set “c2.cp.kegg.v2023.1.Hs.symbols.gmt” as a reference. The clusterProfiler package was utilized to perform Gene Ontology (GO) and Kyoto Encyclopedia of Genes and Genomes (KEGG) pathway enrichment analysis on genes associated with WARS1^30^. GO categories encompassed three different categories, namely biological process (BP), cellular component (CC) and molecular function (MF).

### Construction of diagnostic models

The potential of the 11 progressively altered genes to discriminate among normal, adenoma, and adenocarcinoma samples in PBMC, GSE20916, and GSE117606 datasets was evaluated using Receiver Operating Characteristic (ROC) curves generated with the *pROC* package in R. A random forest algorithm was applied to construct a diagnostic model based on PBMC transcriptome data. The number of trees was set to 100, and the splitting criterion was entropy. To mitigate overfitting, 5-fold cross-validation was performed, and the average accuracy across folds was calculated together with the corresponding ROC curves. This model was used to evaluate the diagnostic potential of the 11 genes in distinguishing disease states. For external validation, the same model parameters were applied to the GSE20916 and GSE117606 datasets to assess reproducibility and discriminatory performance.

### Analysis of clinical information

The clinical data of patients with COAD and READ were retrieved from the TCGA datasets for subsequent analysis. The analysis was conducted on the distribution of 11 progressively altered genes across age, gender, stage and TNM stage.

### Immunohistochemistry

WARS1 expression levels in samples were determined by immunohistochemistry (IHC). Tissues from untreated adenoma and carcinoma patients (no prior chemotherapy, radiotherapy, or medication) were analyzed. This study was approved by the Ethics Committee of Nanjing Hospital of Traditional Chinese Medicine. Tissue sections were incubated with WARS1 antibody (Manufacturer: Abcam; Catalog number: EPR23057-116). To quantify the intensity of WARS1 expression, the average optical density (AOD) value of each sample was calculated using ImageJ software. All samples were independently reviewed by two experienced pathologists who were blinded to sample identity. Inter-observer agreement was assessed, and any discrepancies were resolved by joint review to ensure reproducibility of scoring. Potential confounders such as comorbidities, medications, and lifestyle were not controlled, reflecting real-world clinical conditions.

### Statistical analysis

The statistical analysis was conducted using R software (4.2.2) along with its relevant packages. Group comparisons were performed using the Wilcoxon rank-sum test, for which P-values, effect sizes (rank-biserial correlation, r), and 95% confidence intervals (CI) were calculated. Spearman correlation was applied to evaluate relationships among variables. Statistical significance was determined at *P* < 0.05.

## Results and discussion

### Analysis of transcriptome data of human blood PBMC

**Fig. 1:**
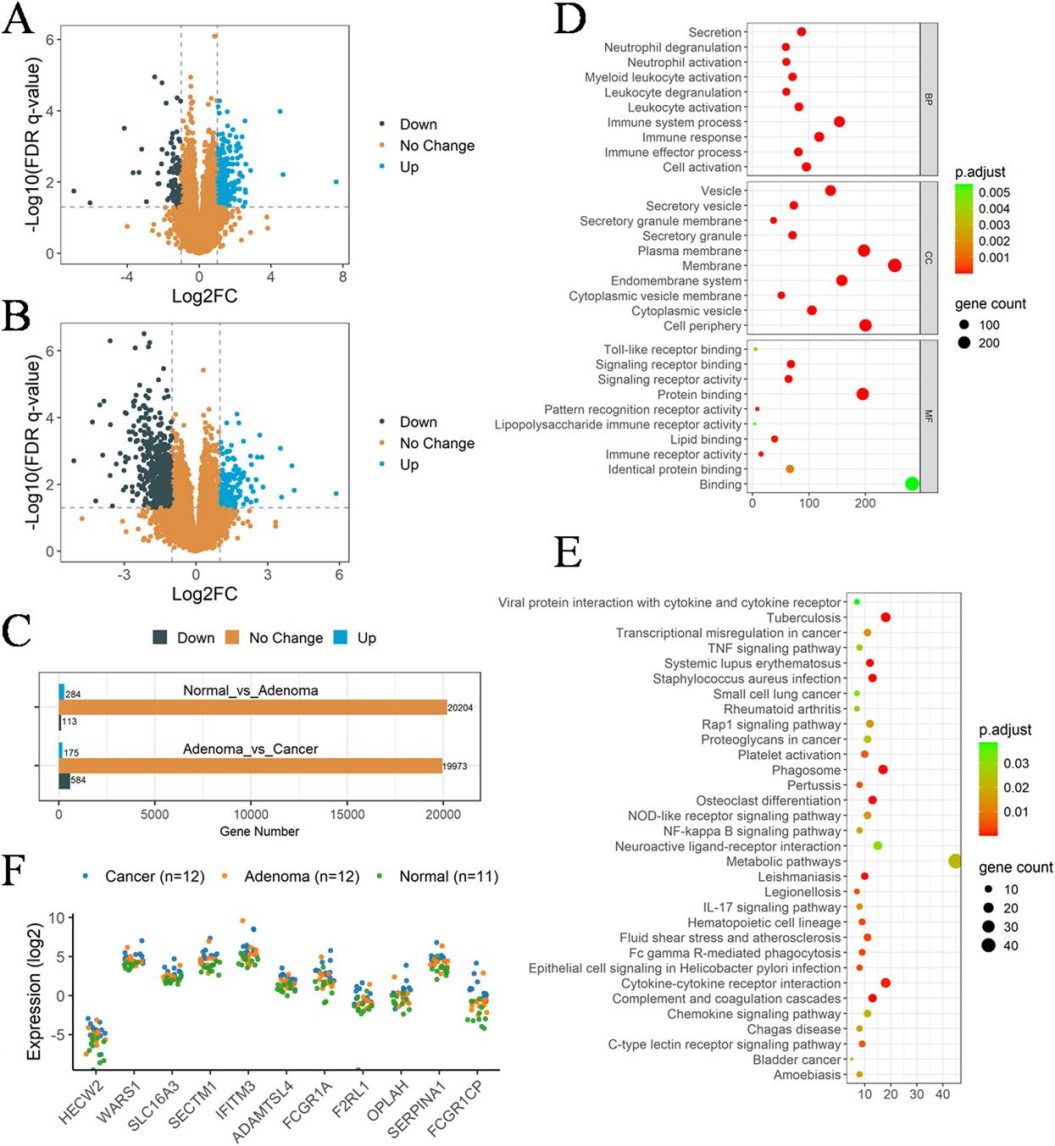
Results of transcriptome data analysis of human blood PBMC. (**A**,** B**) Volcano plot of differential genes. (**A**) Normal (*n* = 11) vs. Adenoma (*n* = 12), (**B**) Adenoma (*n* = 12) vs. Cancer (*n* = 12). (**C**) Distribution statistic of differential genes in different samples. (**D**) GO enrichment pathway of differential genes with continuously change trends. (**E**) KEGG enrichment pathway of differential genes with continuously change trends. (**F**) Expression of 11 differentially expressed genes exhibiting persistent alterations in normal, adenoma and cancer samples, statistical comparisons were performed using the Wilcoxon rank-sum test, and *P* < 0.05 was considered statistically significant. (Normal vs. Adenoma, *p* < 0.05, Adenoma vs. Cancer, *p* < 0.05).

In order to investigate the transcriptomic alterations associated with colorectal cancer progression, we collected peripheral blood samples from subjects across the normal–adenoma–adenocarcinoma sequence. The baseline clinical characteristics of these participants are provided in Table 1. After the transcriptomic data of human blood PBMC were collected, DESeq2 was employed to identify differentially expressed genes between normal and adenoma, as well as between adenoma and cancer. Gene with adjusted P-value ≤ 0.05 and |Log2FC| > 1 found by DESeq2 were assigned as differentially expressed, results were shown in Fig. 1A, B. Compared to the normal group, the adenoma group exhibited 284 up-regulated genes and 113 down-regulated genes, resulting in a total of 397 differentially expressed genes. Compared to the adenoma group, the cancer group exhibited 175 up-regulated genes and 584 down-regulated genes, resulting in a total of 759 differentially expressed genes. The statistical results were shown in Fig. 1C. A list of differentially expressed genes was aggregated, and their mean values were subsequently computed across various groups. The genes exhibiting a continuous increase or decrease in expression were selected based on the progressive order of the “N-A-C” sequence. The enrichment analysis was conducted to unveil the dynamically changing signaling pathways in the “N-A-C” sequence. Based on the findings depicted in Fig. 1D, E, it can be observed that the immune system of the human body remains consistently active throughout the transition from normal to cancer, encompassing immune system process, immune response, immune effector process, leukocyte degranulation, myeloid leukocyte activation, neutrophil activation, etc. The Wilcoxon test was employed to assess the differences in persistently changing genes between different groups, thereby enhancing the precision of the study objectives. Finally, 11 genes showed significant differences in normal and adenoma as well as adenoma and cancer (*p* < 0.05) were identified. The following 11 genes were HECW2, WARS1, SLC16A3, SECTM1, IFITM3, ADAMTSL4, FCGR1A, F2RL1, OPLAH, SERPINA1, FCGR1CP (Fig. 1F).

**Table 1:** The blood PBMC samples corresponding clinical information.

Variable	No.	Normal	Adenoma	I	II	III	IV
Clinicopathologic characteristics							
Age(years)							
≤ 65	22	7	7	1	2	2	3
> 65	13	4	5	1	1	2	0
Gender							
Male	20	6	6	1	2	3	2
Female	15	5	6	1	1	1	1
Location							
Colon	15	—	7	1	3	2	2
Rectum	9	—	5	1	1	1	1
T stage							
T1-3	9	—	—	2	4	2	1
T4	3	—	—	0	0	1	2
N stage							
N0	6	—	—	2	4	0	0
N1-2	6	—	—	0	0	3	3
M stage							
M0	9	—	—	2	4	3	0
M1	3	—	—	0	0	0	3

### Evaluation of the clinical diagnostic value of the 11 progressively altered genes

**Fig. 2:**
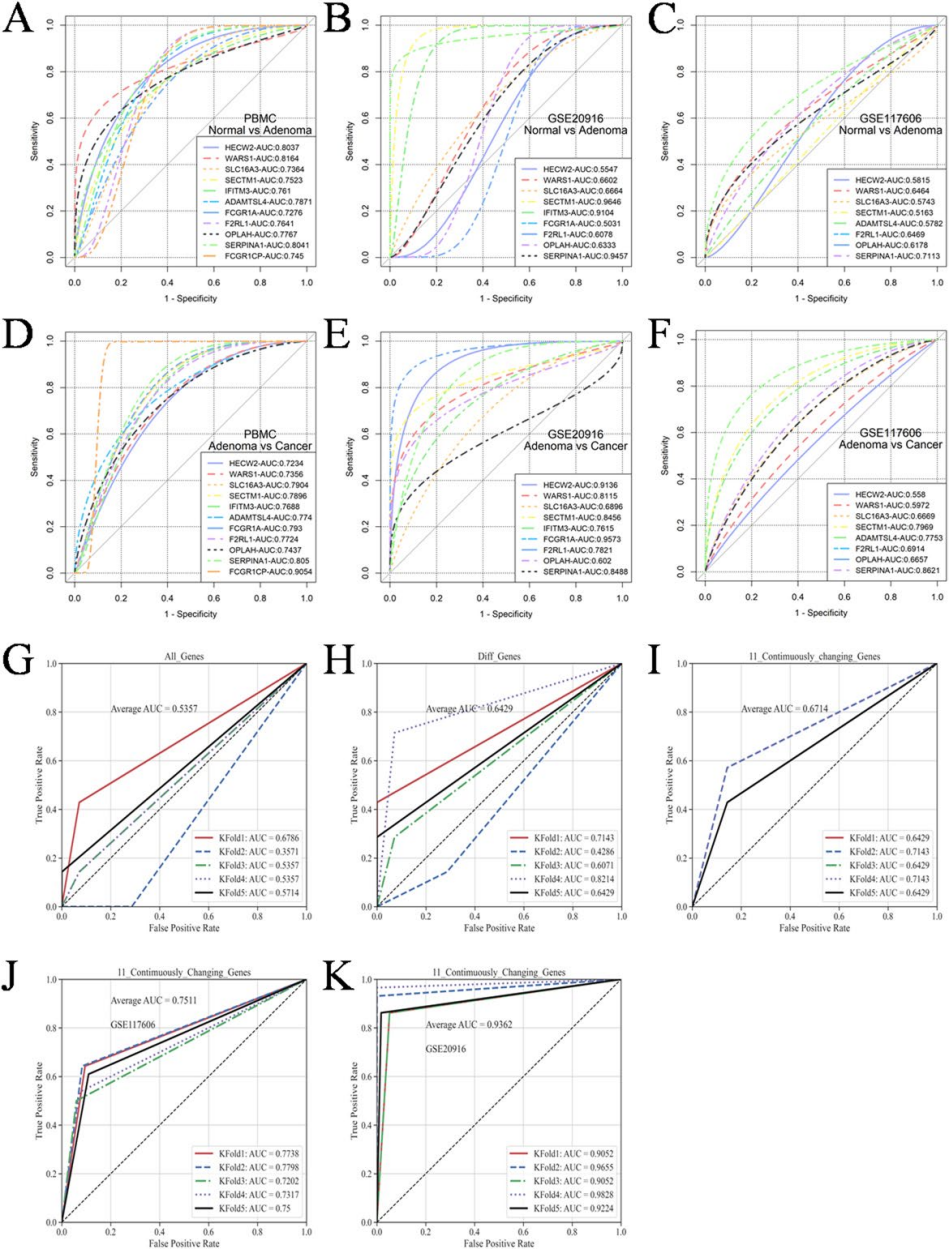
ROC curves for the diagnostic potential of 11 genes. (**A**-**C**) Ability of independent genes in PBMC, GSE117606 and GSE20916 to discriminate between normal and adenoma samples. (**D**-**F**) Ability of independent genes in PBMC, GSE117606 and GSE20916 to discriminate between adenoma and cancer samples. (**G**) ROC of all genes in blood PBMC. (**H**) ROC of differential genes in blood PBMC. (**I**-**K**) ROC of progressively altered genes to discriminate among normal, adenoma and cancer samples.

The roc curves of individual genes were initially generated using the pROC library in the R. The discriminative potential of this curve was evaluated in PBMC, GSE117606 and GSE20916 databases for distinguishing normal, adenoma and cancer samples. The findings demonstrate that 11 genes possess a distinct capability to discriminate between various sample types across different databases (Fig. 2A-F). The identification based on a single gene alone is insufficient for precise identification. To enhance the diagnostic efficacy, it is imperative to incorporate a greater number of genes in the construction of the identification model. Initially, all genes, differentially expressed genes and 11 progressively altered genes were utilized as inputs for training in the case of human blood PBMC samples. The average accuracy obtained from 5-fold cross validation was utilized as the evaluation metric (Fig. 2G, H, I). Accuracy reached its minimum when all genes are utilized as training data, it exhibits a continuous improvement in accuracy with the simplification of gene sets. The presence of excessive redundant feature variables can result in a decline in model accuracy, thereby accounting for the lowest accuracy observed among all genes. Differential gene set eliminated a majority of the non-significantly altered nonsense genes, resulting in an improved accuracy rate of 64.29%. Instead of a decrease, the accuracy was increased to 67.14% when the gene set was reduced to 11, which is quite satisfactory. However, the accuracy demonstrated by these 11 genes was insufficient to establish their diagnostic value, the limited size of the datasets may be one of the contributing factors. For enhancing the evidence of these 11 genes as diagnostic markers, validation was conducted on GSE117606 and GSE20916. The accuracy exhibited a significant improvement on the new datasets, reaching an excitingly high level of 93.62% on GSE20916 (Fig. 2J, K). Overall, the aforementioned findings validate the diagnostic efficacy of these pivotal genes across different sample types. In particular, the 11 progressively altered genes exhibited a remarkable diagnostic performance in the independent tissue-based datasets, reaching an accuracy of 93.62% in GSE20916. These results provide optimism that such genes may serve as promising biomarkers for CRC diagnosis. Nevertheless, since the external validation was performed using tissue-derived datasets rather than blood samples, further confirmation in larger independent PBMC cohorts will be essential before clinical application.

### Comprehensive analysis of expression, genetic and epigenetic alterations and immunological characteristics of progressively altered genes in CRC

**Fig. 3:**
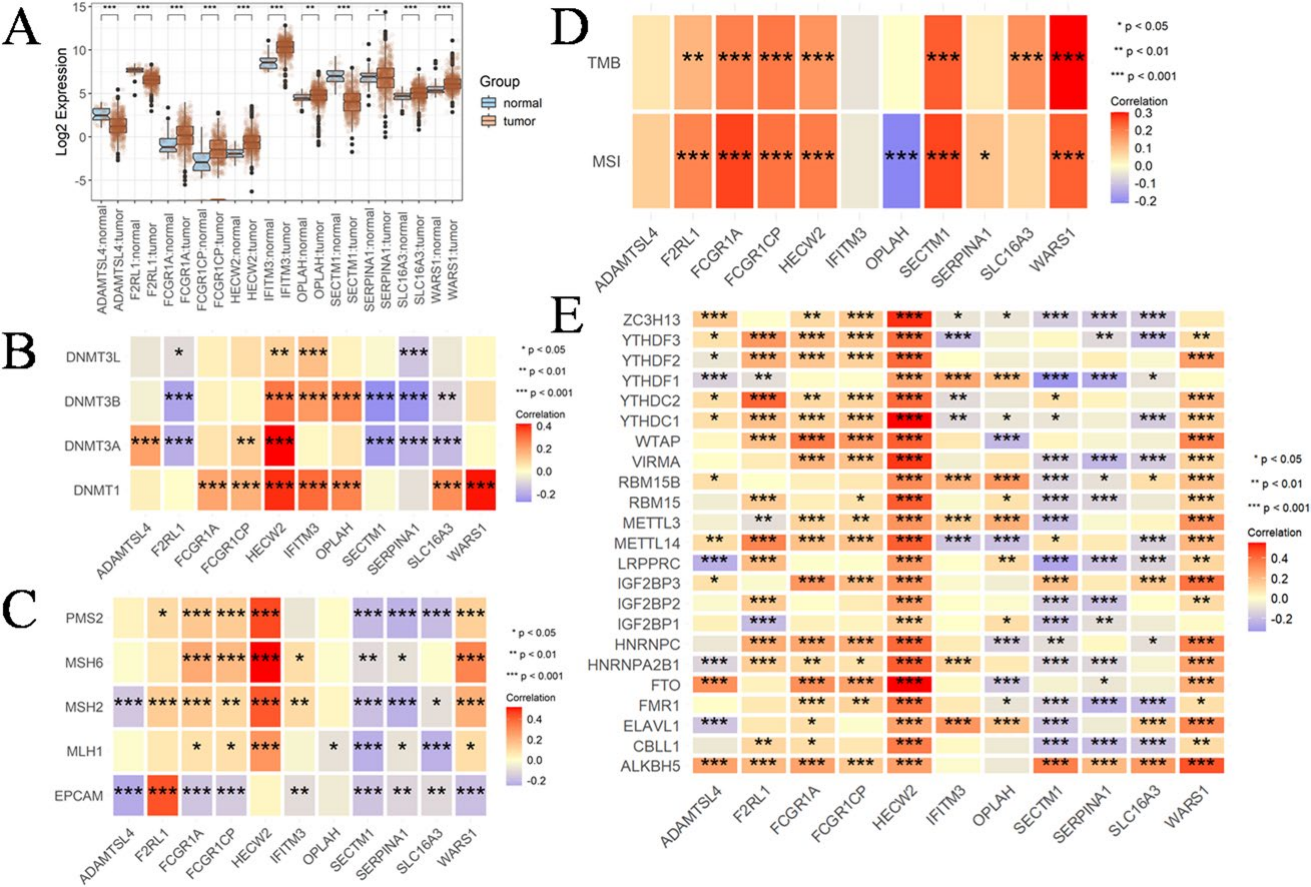
Comprehensive analysis of expression, genetic and epigenetic alterations and immunological characteristics of progressively altered genes (HECW2, WARS1, SLC16A3, SECTM1, IFITM3, ADAMTSL4, FCGR1A, F2RL1, OPLAH, SERPINA1, FCGR1CP) in CRC. (**A**) The differential expression of progressively altered genes between colorectal cancer (*n* = 650) and matched normal specimens (*n* = 51). (**B**) The heatmap effectively visualizes the association between 11 genes and four major DNA methyltransferases, namely DNMT1, DNMT3L, DNMT3A and DNMT3B in colorectal cancer. The first outer ring represents 11 genes, the second ring illustrates four DNA methyltransferases, the third ring displays correction coefficients, the fourth ring presents pvalues and numbers in the inner ring indicate both correlation coefficients and pvalues. (**C**) The heatmap effectively visualizes the association between 11 genes and five DNA mismatch repair genes (MLH1, MSH2, MSH6, PMS2 and EPCAM) in colorectal cancer. (**D**) The heatmap effectively visualizes the association between 11 genes and TMB, MSI in colorectal cancer. (**E**) The heatmap effectively visualizes the association between 11 genes and m6A. Correlation analysis was conducted using Spearman’s rank correlation, and significance was defined as *P* < 0.05. (*P < 0.05, **P < 0.01, ***P < 0.001).

**Fig. 4:**
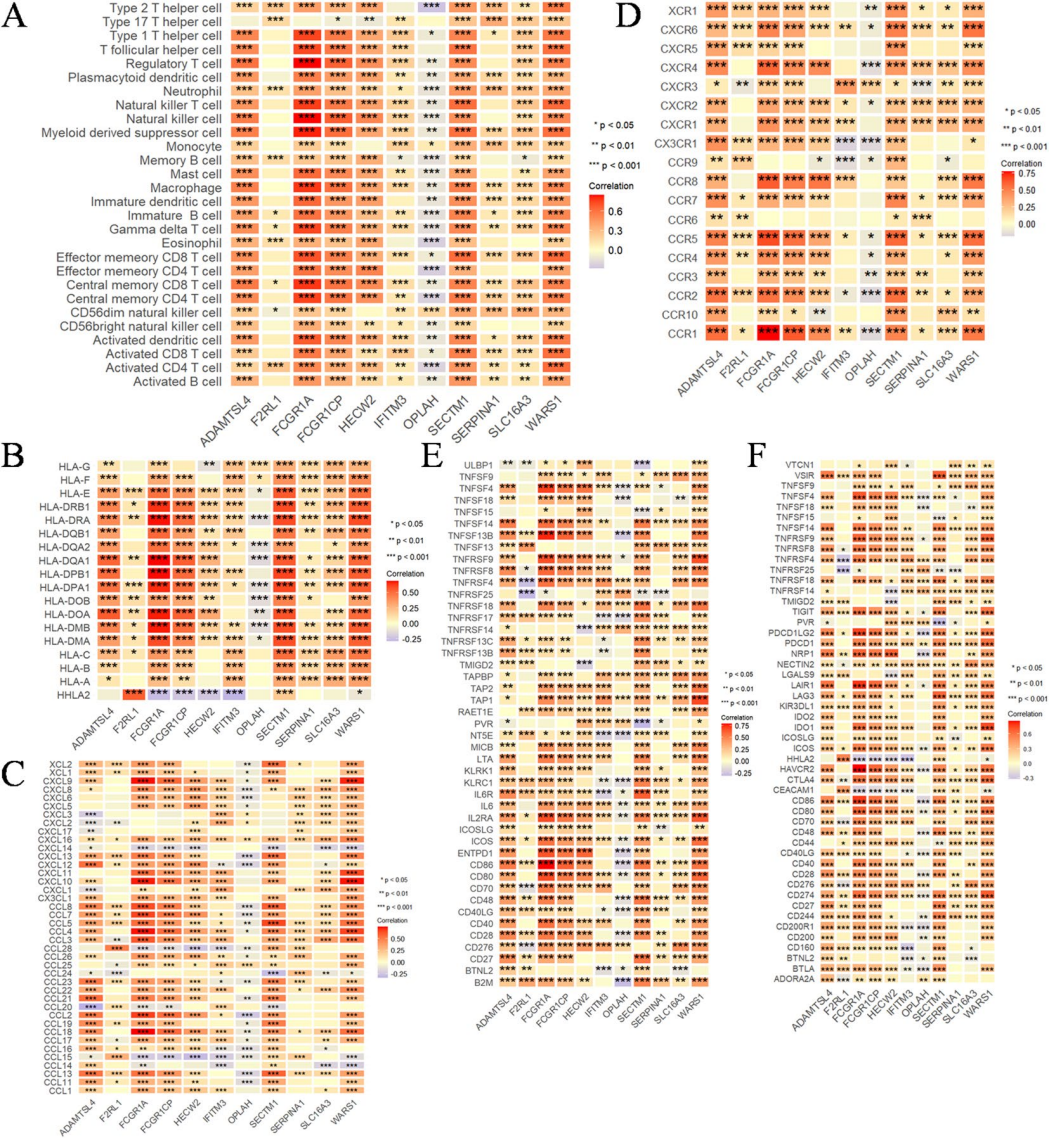
Comprehensive analysis of expression, genetic and epigenetic alterations and immunological characteristics of progressively altered genes (HECW2, WARS1, SLC16A3, SECTM1, IFITM3, ADAMTSL4, FCGR1A, F2RL1, OPLAH, SERPINA1, FCGR1CP) in CRC. (**A**-**F**) Heatmaps visualize the association of 11 genes with (**A**) tumor-infiltrating immune cells, (**B**) MHC molecules, (**C**) chemokines, (**D**) immunostimulatory receptors, (**E**) immunostimulatory factors and (**F**) immune checkpoint molecules in colorectal cancer. Correlation analysis was conducted using Spearman’s rank correlation, and significance was defined as *P* < 0.05. (*P < 0.05, **P < 0.01, ***P < 0.001).

The findings presented in Sect. 3.1 and 3.2 exclusively depict the gene characteristics observed in blood PBMC samples, prompting an inquiry into their involvement in CRC. To further investigate the role of these genes in CRC, the data were obtained from TCGA for analysis. Revealing an unexpected finding, all 11 genes, with the exception of SERPINA1, exhibited a significant change in CRC tissues compared with their corresponding normal tissue (Fig. 3A, Supplementary Table S1). DNA methyltransferases play pivotal roles in modulating chromatin structure and regulating gene expression. We observed a positive correlation among the expression of most of these 11 genes and the four major DNA methyltransferases, namely DNMT1, DNMT3L, DNMT3A and DNMT3B. Although there were negative associations, the observed results showed that the association was weak and did not reach statistical significance. The correlation between WARS1 and DNMT1, as well as HECW2 and DNMT3A, exhibited significant and robust positive associations among the investigated factors (Fig. 3B). The results depicted in Fig. 3C demonstrate significant positive correlations among HECW2-MSH2, HECW2-MSH6, HECW2-PMS2, WARS1-MSH6, and F2RL1-EPCAM. The TMB emerges as a robust biomarker for predicting responsiveness to immune checkpoint blockade^31^. In Fig. 3D, HECW2, WARS1, SLC16A3, SECTM1, FCGR1A, F2RL1 and FCGR1CP exhibited a significant positive correlation with TMB. The hypermutation phenotype of MSI arises from frequent polymorphisms in repetitive DNA sequences, as well as single nucleotide substitutions resulting from defects in DNA MMR^32^. We observed positive associations between HECW2, WARS1, SECTM1, FCGR1A, F2RL1, SERPINA1 and FCGR1CP with MSI and negative associations with OPLAH in CRC. However, these correlations exhibited a generally weak association. The most prevalent RNA modification, exerts a profound impact on the intricacy of cancer progression^33^. In this study, our focus was on investigating the correlation between 11 genes and m6A regulators in CRC. In Fig. 3E, we should prioritize our attention towards HECW2, WARS1 and SECTM1 due to their significant associations with the majority of m6A regulators. Subsequently, we conducted an immunological characterization of these genes in CRC. Apart from OPLAH, the remaining genes exhibited a significant positive correlation with immune cells (Fig. 4A). Likewise, they exhibited significant correlations with other factors that influence the immune system. Our data suggested that the 11 genes were remarkably positively correlated to MHC molecules (Fig. 4B), chemokines (Fig. 4C), immunostimulatory receptors (Fig. 4D), immunostimulatory factors (Fig. 4E) and immune checkpoint molecules (Fig. 4F) in CRC.

The occurrence and development of tumors involve a multi-step process encompassing genetic and epigenetic alterations in both tumor cells and normal cells^34,35^. The nosogenesis of CRC involve mechanisms such as chromosome instability, high MSI, and methylation, which contribute to the mutation of oncogenes, tumor suppressor genes and mismatch repair genes^36^. Our findings provide compelling evidence for the pivotal roles of these genes in the pathogenesis of CRC.

### Analysis of clinical information for progressively altered genes

**Fig. 5:**
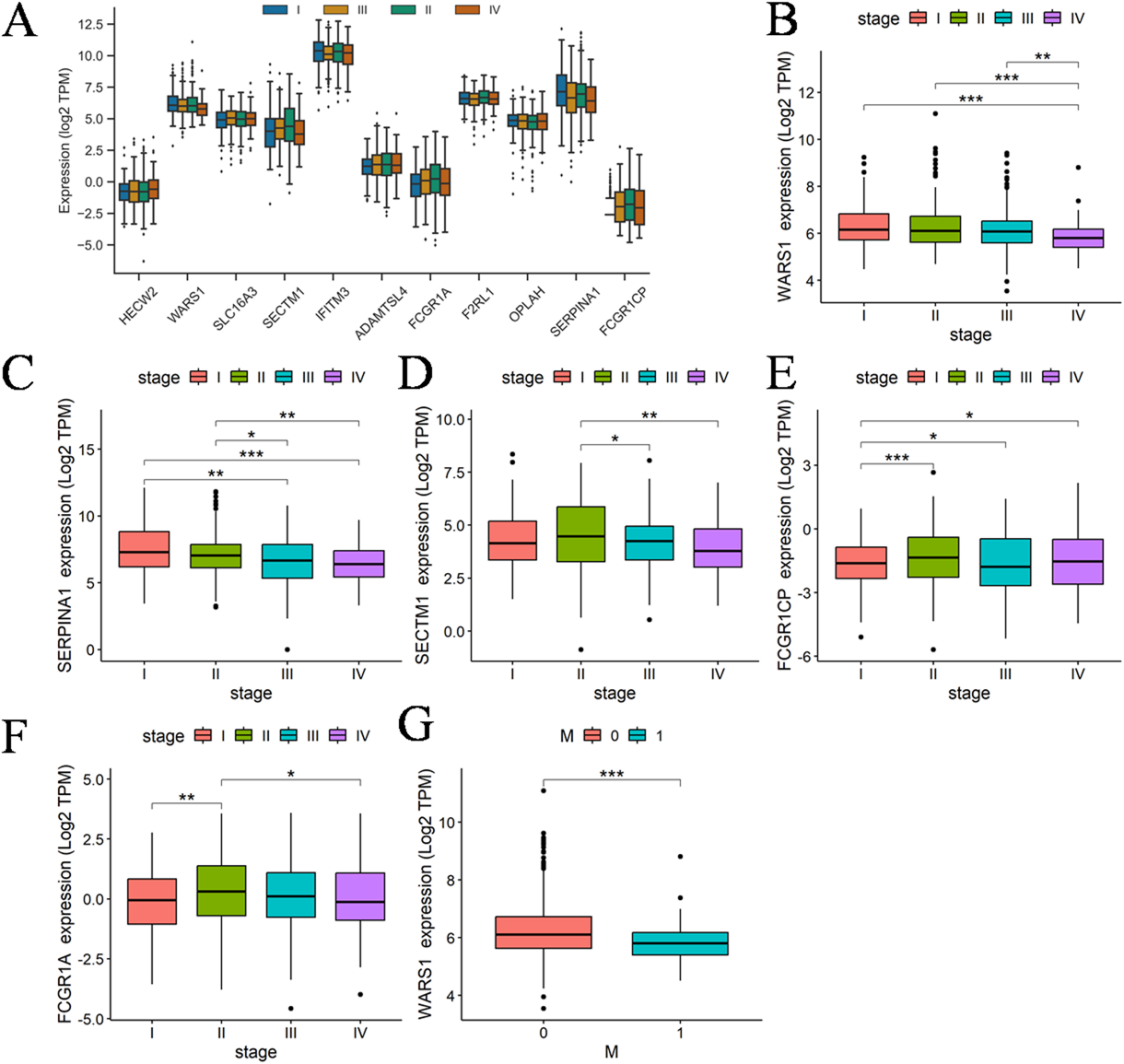
Results of clinical correlation analysis. (**A**) The expression of 11 genes in different clinical stages was visualized using boxplots (93 patients in stage I, 210 patients in stage II, 147 patients in stage III, and 86 patients in stage IV). (**B**-**E**) Boxplots illustrate the differential expression of genes across distinct clinical stages, the p-values of the wilcoxon test for different stages are annotated on the picture, (**B**) WARS1, (**C**) SERPINA1, (**D**) SECTM1, (**E**) FCGR1CP, (**F**) FCGR1A. (**G**) Expression of WARS1 in M0 and M1 samples. Statistical comparisons were performed using the Wilcoxon rank-sum test, and *P* < 0.05 was considered statistically significant. (*P < 0.05, **P < 0.01, ***P < 0.001).

The “N-A-C” sequence represents the cascade of events leading to cancer initiation, while the subsequent stages following cancer initiation encompass the process of cancer progression. Consequently, it is anticipated that there will be a consistent and directional alteration in the expression of these genes across distinct clinical stages. The expression patterns of 11 genes in various clinical stages are depicted in Fig. 5A. The results revealed that only WARS1, SERPINA1, SECTM1 and FCGR1CP exhibited statistically significant alterations across different stages. The expression details were illustrated in Fig. 5B-G. The statistical parameters of the wilcoxon test are presented in Supplementary Table 2. SECTM1 and FCGR1CP exhibited significant different expression without adhering to a discernible pattern. Reassuringly, the expression of WARS1 and SERPINA1 exhibit a progressive decline throughout the course of cancer. However, there was no significant difference in SERPINA1 expression between normal and cancer tissues, so we conducted a comprehensive analysis of WARS1. Importantly, our results indicate that WARS1 expression is significantly elevated in the cancer group compared with both normal and adenoma, and this pattern was consistently observed in both blood-derived PBMC and colorectal tissue samples along the normal–adenoma–carcinoma sequence. Thus, WARS1 shows potential in distinguishing cancer from precancerous lesions. However, within the cancer stage itself, analysis of the TCGA cohort revealed a gradual decrease in WARS1 expression from stage I to stage IV, suggesting that while WARS1 is upregulated during carcinogenesis, it subsequently declines with disease progression, possibly reflecting immune suppression and metastatic evolution.

The TNM staging, another clinical indicator of WARS1, exhibited significant differences between M0 and M1 (Fig. 5G). The clinical data from the TCGA database indicates that distant metastasis was exclusively observed in patients diagnosed with stage 4, thereby suggesting that M1 classification was solely appeared at stage 4. Remarkably, the expression of WARS1 exhibited a gradual decline in tandem with the advancement of CRC, reaching its nadir at stage 4. This trend coincides with the alterations in the immune system throughout the onset and progression of cancer, transitioning from an initial immune burst to immune evasion, ultimately culminating in tumor metastasis.

### Trends in progressively altered genes in intestinal tissue

**Fig. 6:**
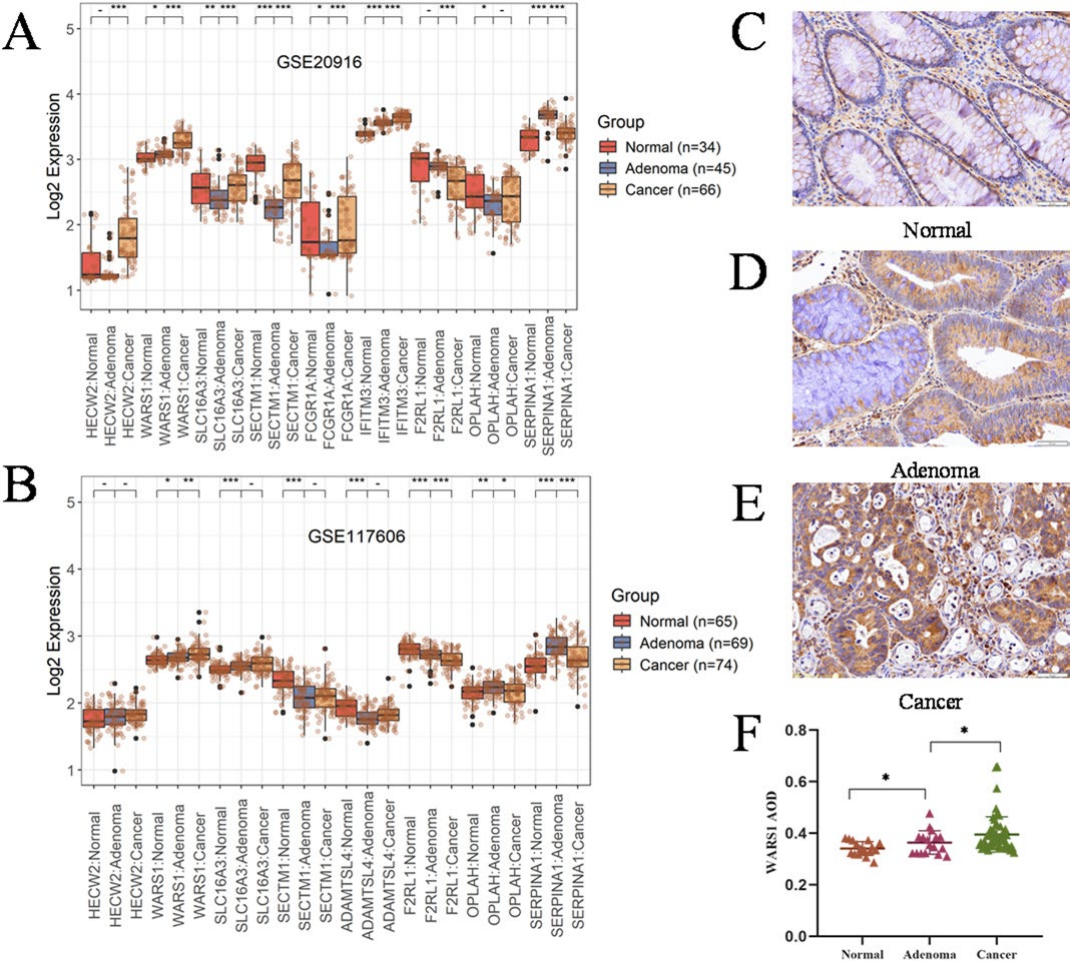
Expression of progressively altered genes in intestinal tissue. (**A**) Expression of WARS1 in normal (*n* = 34), adenoma (*n* = 45), and cancer (*n* = 66) in the GSE20916 dataset. (**B**) Expression of WARS1 in normal (*n* = 65), adenoma (*n* = 69), and cancer (*n* = 74) in the GSE117606 dataset. (**C**-**E**) Comparison of WARS1 expression in normal, adenoma and colorectal cancer by IHC. (**F**) AOD of WARS1 in normal (*n* = 17), adenoma (*n* = 17) and colorectal cancer (*n* = 58). Statistical comparisons were performed using the Wilcoxon rank-sum test, and *P* < 0.05 was considered statistically significant. (*P < 0.05, **P < 0.01, ***P < 0.001).

The trend of 11 genes that exhibited continuous upregulation in PBMC was investigated using two datasets, namely GSE20916 and GSE117606. Out of these 11 genes, only WARS1, IFITM3 and SLC16A3 exhibited a consistent pattern with PBMC in Fig. 6A and B. The statistical parameters of the wilcoxon test are presented in Supplementary Table 3. The expression levels of WARS1 and IFITM3 exhibited significant variations among normal, adenoma and cancer tissues. A limitation of this study is that not all of the 11 genes were represented in both datasets.

The role of IFITM3 in CRC has been documented, whereas the association between WARS1 and CRC remains ambiguous. Subsequently, our investigation will be directed towards elucidating the association between WARS1 and CRC. The immunohistochemical study was conducted using 93 cases of pathological tissue sections, including normal, adenoma, different clinical staging CRC and metastasis, collected from clinical sources. The clinical information statistics are presented in Table 2. Figure 6C-E display immunohistochemical sections of normal intestinal tissue, adenomatous tissue, and cancerous tissue, respectively. The statistical results of IHC are presented in Fig. 6F, demonstrating a progressive significant increase in the expression of WARS1 along the “N-A-C” sequence. The statistical parameters of the wilcoxon test are presented in Supplementary Table 4. In addition to strong epithelial expression of WARS1 in adenoma and carcinoma cells (Fig. 6), previous studies have shown that WARS1 can also be secreted or induced in immune cells. It can activate TREM-1 signaling in macrophages and be released into the extracellular environment by monocytes under pathogenic stimuli, and it can even be secreted without requiring de novo synthesis^45–47^. These findings suggest that the elevated WARS1 levels in PBMC may result from both tumor-derived release and immune cell activation, reconciling its upregulation in both tissue and blood samples.

**Table 2:** The pathological tissue slice corresponding clinical information.

Variable	No.	Normal	Adenoma	I	II	III	IV	Metastasis
Clinicopathologic characteristics								
Age(years)								
≤ 65	51	11	8	8	5	6	5	8
> 65	42	6	9	5	9	4	5	4
Gender								
Male	55	10	9	9	9	5	7	6
Female	38	7	8	4	5	5	3	6
Location								
Colon	41	10	12	4	8	5	2	—
Rectum	40	7	5	9	6	5	8	—
T stage								
T1-3	38	—	—	13	7	7	5	6
T4	21	—	—	0	7	3	5	6
N stage								
N0	27	—	—	13	14	0	0	0
N1-2	32	—	—	0	0	10	10	12
M stage								
M0	37	—	—	13	14	10	0	0
M1	22	—	—	0	0	0	10	12

### Immunological and biological significance of WARS1 in CRC

**Fig. 7:**
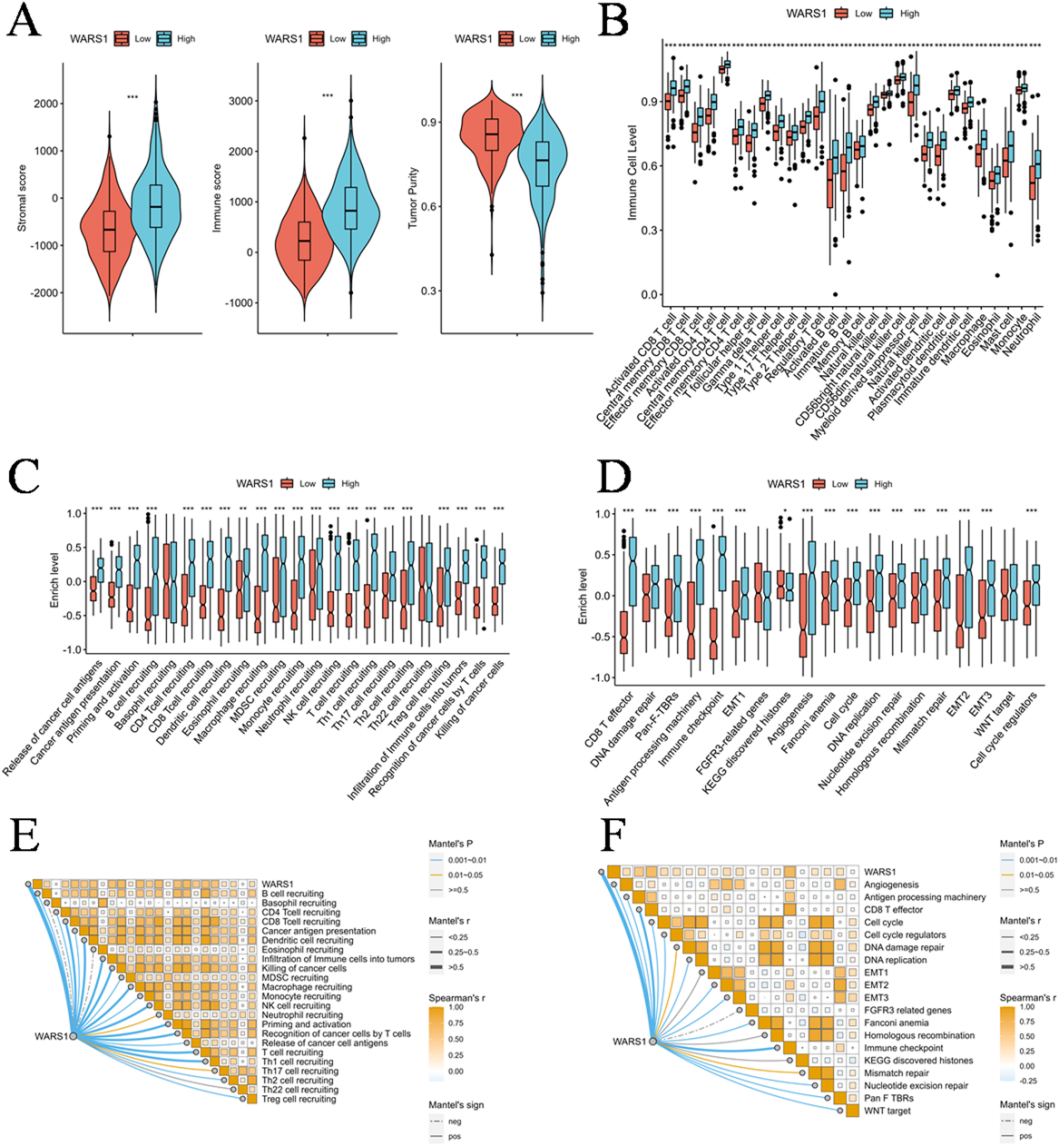
Immunological and biological significance of WARS1 in colorectal cancer. (**A**) Assessment of disparities in stromal score, immune score and tumor purity between subgroups with high and low WARS1 expression. (**B**) Comparative analysis of immune cell infiltration between subgroups with high and low WARS1 expression levels. (**C**) Quantification of the cancer immunity cycle activities in two distinct subgroups. (**D**) Revealing differential activation of known biological signatures in two distinct subgroups. (**E**) Associations of WARS1 expression with the cancer immunity cycle activities. (**F**) Associations of WARS1 expression with the known biological signatures. Statistical comparisons were performed using the Wilcoxon rank-sum test, and *P* < 0.05 was considered statistically significant. Correlation analysis was conducted using Spearman’s rank correlation, and significance was defined as *P* < 0.05. (*P < 0.05, **P < 0.01, ***P < 0.001).

Immunohistochemistry demonstrated strong WARS1 staining in epithelial cells of adenoma and carcinoma lesions (Fig. 6C-E). In contrast, ESTIMATE analysis of TCGA CRC transcriptomes revealed that WARS1 expression was positively associated with stromal and immune scores and negatively correlated with tumor purity (Fig. 7A, Supplementary Table S5). This difference reflects both disease stage and cellular context. During the adenoma–carcinoma transition, WARS1 is elevated in epithelial cells, marking early carcinogenesis. However, in established CRC (stages I–IV), its expression declines in parallel with progression, consistent with immune evasion and metastatic potential. At the same time, bulk transcriptomic profiles integrate signals from non-malignant compartments. Previous studies have demonstrated that WARS1 can be induced and secreted by immune cells such as macrophages and monocytes^45,46^, while CAFs are well-established stromal regulators that remodel the microenvironment and influence immune responses^48^. Thus, the apparent paradox between IHC and TCGA results can be explained by a dual-phase and dual-source model, where WARS1 reflects epithelial transformation in early carcinogenesis and immune-stromal alterations during cancer progression.

Consistent with this dual context, ssGSEA revealed significantly increased infiltration of all analyzed immune cell population in the subgroup with high WARS1 expression (Fig. 7B, Supplementary Table S6). Enhanced activities of most steps within the cancer immunity cycle were also observed in this subgroup (Fig. 7C, Supplementary Table S7). Furthermore, WARS1-high tumors exhibited augmented activities of cell cycle regulation, DNA repair pathways, and immune activation processes (e.g., CD8 + T effector function, antigen processing machinery, immune checkpoints), as well as stromal activation programs including EMT, angiogenesis, and fibroblast signatures (Fig. 7D, Supplementary Table S8). The correlations between WARS1 expression and both the cancer immunity cycle and stromal/immune activation processes (Fig. 7E, F) therefore echo the results from ESTIMATE and support the view that WARS1 reflects not only epithelial transformation but also tumor microenvironment remodeling. Taken together, while the involvement of WARS1 in innate immunity has been established^37^, our findings extend its significance to a dual role: a marker of epithelial transformation during early carcinogenesis and an indicator of immune-stromal alterations in established CRC.

### Association of WARS1 with mutational landscape in CRC

**Fig. 8:**
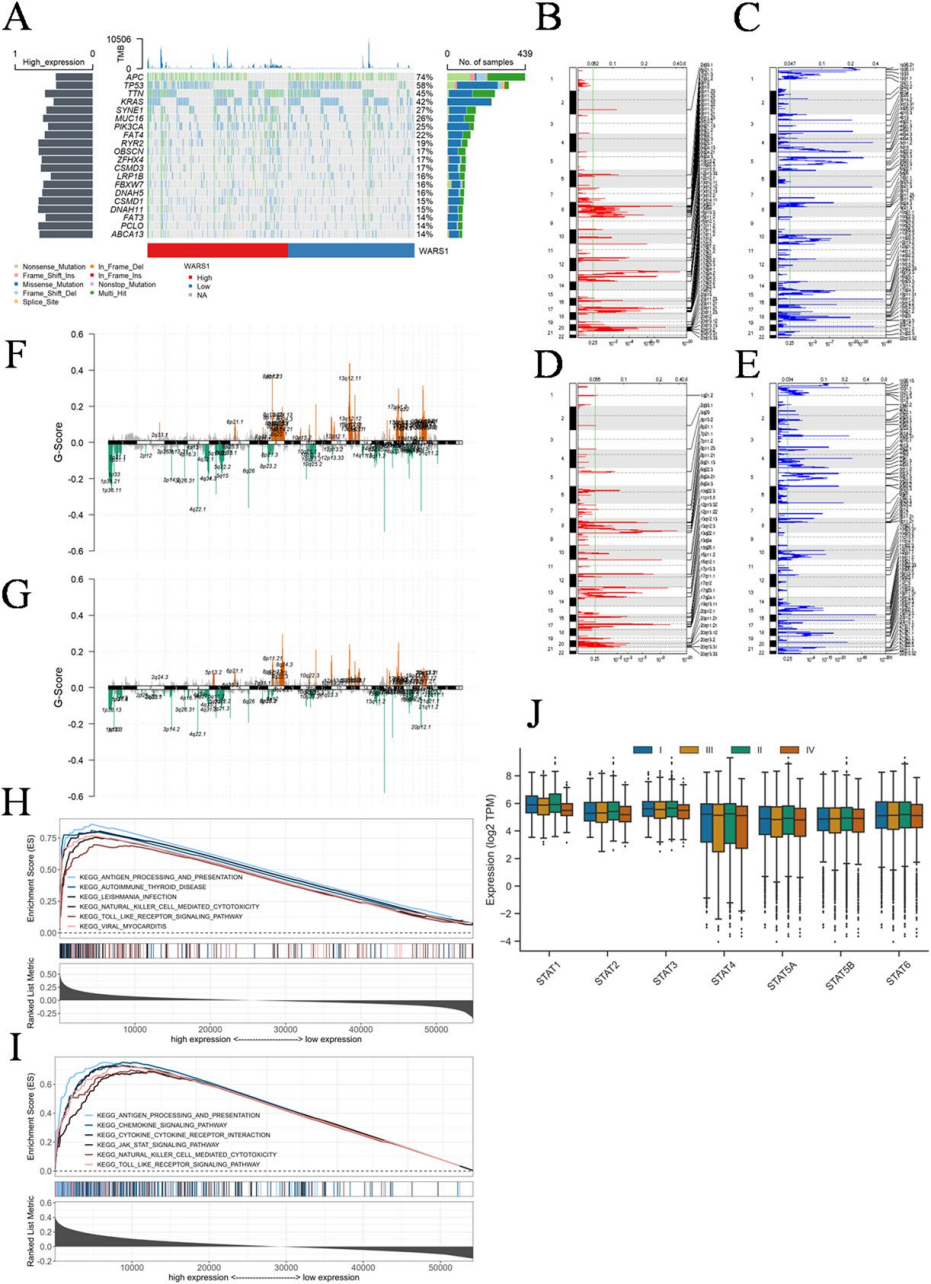
Association of WARS1 with mutational landscape and signaling pathway in CRC individuals. (**A**) Landscape of somatic mutation in high and low WARS1 expression subgroups of CRC. Genes are ranked based on frequency of mutation. The upper section of the chart presents the TMB scores for each patient. The right barplot illustrates the distribution of samples based on gene mutation types. The left barplot illustrates the distribution of gene mutations in high WARS1 expression subgroup. (**B**,** C**) Landscape of (**B**) amplification and (**C**) deletion in high WARS1 expression subgroup. (**D**,** E**) Landscape of (**D**) amplification and (**E**) deletion in low WARS1 expression subgroup. The genome is vertically oriented from top to bottom. The GISTIC 2.0 qvalue at each locus is a left-to-right manner. The green line represents the threshold value of qvalue = 0.25. (**F**,** G**) Detection and comparison of copy number amplification and deletion in subgroups with (**F**) high and (**G**) low expression levels of WARS1. (**H**) GSEA annotation of activated pathway in high WARS1 expression subgroup. (**I**) GSEA annotation of activated pathway in low WARS1 expression subgroup. (**J**) The expression pattern of STAT family genes undergoes dynamic changes across different development stages.

In Fig. 8A, we assessed the prevalence of somatic mutations in subgroups with high and low WARS1 expression levels. The findings revealed the top 20 genes with significantly elevated mutation rates. The WARS1 high expression subgroups exhibited a significant increase in mutations across the majority of genes, with more than 50% of the population affected. The GISTIC2.0 results revealed a higher frequency of amplification and deletion in the subgroup with high WARS1 expression (Fig. 8B, C) compared to the subgroup with low WARS1 expression (Fig. 8D, E). Moreover, the G-score was computed by considering both the magnitude of aberrations and their frequency of occurrence in CRC specimens. Following the comparison, a higher number of mutations were observed in multiple regions within the subgroup exhibiting high expression levels of WARS1 (Fig. 8F, G). The mutation on chromosome 13q12.11 (IL7R) exhibited a higher level of significance in the low expression subgroup compared the high expression subgroup. The IL7R gene encodes a receptor for interleukin-7 (IL7), which plays a crucial role in the normal development and homeostasis of T cells^38,39^. Mice deficient in IL7 or IL7R exhibit an early impediment in thymocyte development and a diminished population of non-functional peripheral T cells^40,41^. The presence of IL7R-inactivating mutations leads to the development of severe combined immunodeficiency in humans^42,43^. Immunodeficiency resulting from IL7R gene mutation in the WARS1 low expression subgroup may constitute a significant factor contributing to the relentless progression and metastasis of CRC.

### Signaling pathway associated with WARS1

We conducted a comprehensive analysis of the signaling pathways associated with WARS1 using GSEA. In Fig. 8H, high WARS1 expression was positively correlated to antigen processing and presentation, autoimmune thyroid disease, leishmania infection, natural killer cell mediated cytotoxicity, toll-like receptor signaling pathway, viral myocarditis. Low WARS1 expression, similarly, was positively correlated to antigen processing and presentation, chemokine signaling pathway, cytokine-cytokine receptor interaction, JAK-STAT signaling pathway, natural killer cell mediated cytotoxicity, toll-like receptor signaling pathway (Fig. 8I). The majority of activated pathways in both subgroups were found to be immune-related. However, the activation of the JAK-STAT pathway in the low expression subgroup warrants our attention as it is a dual-edged sword in cancer. While several members of the STAT family, including STAT1, STAT2, STAT3, STAT4, STAT5A, STAT5B and STAT6, have been implicated in tumor initiation and progression (STAT3 and STAT5), others play crucial roles in antitumor defense and the maintenance of an effective long-term immune response (STAT1 and STAT2) through evolutionarily conserved mechanisms^44^. The expression of WARS1 is comparatively lower in CRC subgroup with stage III and IV, while immune-related STAT1 and STAT2 exhibit a decrease, which is associated with tumor growth mediated by STAT3 and 5 have no significant changes (Fig. 8J). Therefore, we are inclined to believe that the activation of the JAK-STAT pathway in the subgroup with low expression of WARS1 contributes significantly to the growth and metastasis of CRC.

## Conclusion

This study systematically examined gene expression changes across the normal–adenoma–carcinoma (N–A–C) sequence of colorectal cancer (CRC) using blood-derived and tissue-based datasets. We identified 11 progressively altered genes in PBMC and validated their diagnostic relevance across independent cohorts. At the systems level, this gene set showed consistent associations with DNA methyltransferases, mismatch-repair genes, m^6A regulators, and genomic-instability markers such as TMB and MSI, and the high-expression subgroup exhibited more frequent copy-number alterations. These results suggest that the panel reflects not only diagnostic potential but also links to genomic regulation and instability.

Among these genes, WARS1 emerged as the most consistent and biologically informative candidate. WARS1 was upregulated during the N–A–C transition and strongly expressed in epithelial cells by IHC, suggesting a role in early carcinogenesis. In established CRC, however, WARS1 expression declined from stage I to IV and correlated positively with stromal and immune scores while inversely with tumor purity, highlighting its link to microenvironmental remodeling.

Analysis of the mutational landscape revealed that WARS1-high tumors harbored elevated TMB and MSI levels as well as a higher frequency of copy-number alterations, supporting its association with genomic instability. In parallel, pathway enrichment showed that WARS1 expression was related to enhanced immune-cell infiltration, activation of the cancer immunity cycle, and upregulation of cell-cycle and DNA-repair pathways, together with stromal programs including EMT and angiogenesis.

Taken together, these findings support a dual role for WARS1: as a marker of epithelial transformation in precancerous lesions, and as a contributor to genomic instability and immune–stromal remodeling during CRC progression. WARS1 therefore represents both a promising diagnostic biomarker and a mechanistically relevant factor in CRC pathogenesis, warranting further validation in large blood-based cohorts and functional studies.

## Supplementary Information

Below is the link to the electronic supplementary material.

Supplementary Material 1

## Data Availability

The RNA-sequence data generated by Reumers J can be accessed on the GEO database with the accession number GSE117606. The processed data from Skrzypczak M’s RNA-sequence study is accessible through the GEO portal under accession number GSE20916. The RNA-sequence data for TCGA and relevant clinical data are obtainable from https://www.cancer.gov/ccg/research/genome-sequencing/tcga.

## Abbreviations

CRC: Colorectal cancer
N-A-C: Normal-adenoma-cancer
PBMC: Peripheral blood mononuclear cell
TCGA: The cancer genome atlas
HECW2: HECT, C2 and WW domain containing E3 ubiquitin protein ligase 2
WARS1: Tryptophanyl-tRNA synthetase 1
SLC16A3: Solute carrier family 16 member 3
SECTM1: Secreted and transmembrane 1
IFITM3: Interferon induced transmembrane protein 3
ADAMTSL4: ADAMTS-like 4
FCGR1A: Fc gamma receptor Ia
F2RL1: F2R like trypsin receptor 1
OPLAH: 5-oxoprolinase
SERPINA1: Serpin family A member 1
FCGR1CP: Fc gamma receptor Ic
TMB: Tumor mutational burden
MSI: Microsatellite instability
APC: Adenomatous polyposis coli
EGFR: Epidermal growth factor receptor
P53: Cellular tumor antigen p53
SMAD4: SMAD family member 4
TGF-β: Transforming growth factor beta
GSVA: Gene set variation analysis
ssGSEA: Single sample gene set enrichment analysis
m6A: N6-methyladenosine
EPCAM: Epithelial cell adhesion molecule
PMS2: PMS1 homolog 2
MSH2: DNA mismatch repair protein 2
MSH6: DNA mismatch repair protein 6
MLH1: DNA mismatch repair protein Mlh1
YTHDF1-3: YTH N6-methyladenosine RNA binding protein F1-3
YTHDC1-2: YTH N6-methyladenosine RNA binding protein C1-2
LRPPRC: Leucine rich pentatricopeptide repeat containing
IGF2BP1-3: Insulin-like growth factor 2 mRNA binding protein 1–3
HNRNPC: Heterogeneous nuclear ribonucleoprotein C
HNRNPA2B1: Heterogeneous nuclear ribonucleoprotein A2/B1
FMR1: Fragile X messenger ribonucleoprotein 1
ELAVL1: ELAV like RNA binding protein 1
FTO: FTO alpha-ketoglutarate dependent dioxygenase
ALKBH5: AlkB homolog 5
ZC3H13: Zinc finger CCCH-type containing 13
WTAP: WT1 associated protein
RBM15/15B: RNA binding motif protein 15/15B
METTL3: Methyltransferase-like 3
METTL14: Methyltransferase-like 14
VIRMA: Vir like m6A methyltransferase associated
CBLL1: Cbl proto-oncogene like 1
DNMT1/2/3A/3B: DNA methyltransferase 1/2/3 alpha/3 beta
COAD: Colon adenocarcinoma
READ: Rectum adenocarcinoma
MHC: Major histocompatibility complex
GEO: Gene expression omnibus
CNV: Copy number variations
KEGG: Kyoto encyclopedia of genes and genomes
GO: Gene ontology
EMT1-3: Erythritol mannosyl transferase 1–3
Pan-F-TBRS: Pan-fibroblast TGF-β response signature
CD8 + T: CD8 + T cell
BP: Biological process
CC: Cellular component
MF: Molecular function
ROC: Receiver operating characteristic curve
TNM: Tumor node metastasis
IL7R: Interleukin 7 receptor
STAT1-4,5A/5B,6: Signal transducer and activator of transcription 1–4,5A/5B,6
JAK-STAT: Janus tyrosine kinase signal transducer and activator of transcription

## References

[1] Pathak, P. S., Chan, G., Deming, D. A. & Chee, C. E. State-of-the-Art management of colorectal cancer. *ASCO Educ. Book.* **44**, 1–15. 10.1200/EDBK_438466 (2024). 10.1200/EDBK_438466 38768405

[2] De Angelis, R. et al. Complete cancer prevalence in Europe in 2020 by disease phase: prevalence, mortality, and trends. *Lancet Oncol.* **25** (2), 123–135. 10.1016/S1470-2045(23)00646-0 (2024). 10.1016/S1470-2045(23)00646-0 38307102

[3] Roshandel, G. et al. Colorectal cancer: Epidemiology, risk factors, and prevention. *Cancers* **16** (8), 1530. 10.3390/cancers16081530 (2024). 38672612 10.3390/cancers16081530 PMC11049480

[4] Wong, S. H. & Yu, J. Gut microbiota in colorectal cancer: mechanisms of action and clinical applications. *Nat. Rev. Gastroenterol. Hepatol.* **16** (11), 690–704. 10.1038/s41575-019-0209-8 (2019). 31554963 10.1038/s41575-019-0209-8

[5] Strum, W. B. Colorectal adenomas. *N. Engl. J. Med.* **374** (11), 1065–1075. 10.1056/NEJMra1513581 (2016). 26981936 10.1056/NEJMra1513581

[6] Kim, H. M. et al. Screening and surveillance for hereditary colorectal cancer syndromes. *Intest Res.* **22** (1), 60–72. 10.5217/ir.2023.00112 (2024). 10.5217/ir.2023.00112 PMC11079514 38311713

[7] Brandaleone, L. et al. Hereditary colorectal cancer syndromes and inflammatory bowel diseases: risk management and surveillance strategies. *Cancers* **16** (17), 2967. 10.3390/cancers16172967 (2024). 39272825 10.3390/cancers16172967 PMC11394661

[8] Bettington, M. et al. The serrated pathway to colorectal carcinoma: current concepts and challenges. *Histopathology* **63** (3), 367–386. 10.1111/his.12055 (2013). 10.1111/his.12055 23339363

[9] Hewish, M., Lord, C. J., Martin, S. A., Cunningham, D. & Ashworth, A. Mismatch repair deficient colorectal cancer in the era of personalized treatment. *Nat. Rev. Clin. Oncol.* **7** (4), 197–208. 10.1038/nrclinonc.2010.18 (2010). 20177404 10.1038/nrclinonc.2010.18

[10] Nielsen, J. C., Ploug, M., Baatrup, G. & Kroijer, R. Risk of post-colonoscopy colorectal cancer following screening colonoscopy with low-risk or no adenomas: a population-based study. *Colorectal Dis.* **23** (3), 630–643. 10.1111/codi.15886 (2021). 10.1111/codi.15886 34427981

[11] Dekker, E., Tanis, P. J., Vleugels, J. L. A., Kasim, P. M. & Wallace, M. B. Colorectal cancer. *Lancet* **394**, 1467–1480. 10.1016/S0140-6736(19)32319-0 (2019). 31631858 10.1016/S0140-6736(19)32319-0

[12] Guo, M. & Schimmel, P. Essential nontranslational functions of tRNA synthetases. *Nat. Chem. Biol.* **9**, 145–153. 10.1038/nchembio.1158 (2013). 23416400 10.1038/nchembio.1158 PMC3773598

[13] Park, S. G., Schimmel, P. & Kim, S. Aminoacyl tRNA synthetases and their connections to disease. *Proc. Natl. Acad. Sci. USA*. **105** (32), 11043–11049. 10.1073/pnas.0802862105 (2008). 18682559 10.1073/pnas.0802862105 PMC2516211

[14] Puleo, A., Carroll, C., Maecker, H. T. & Gupta, R. Isolation of peripheral blood mononuclear cells using Vacutainer^®^ cellular Preparation tubes (CPT™). *Bio Protoc.* **7** (2), e2103. 10.21769/BioProtoc.2103 (2017). 34458433 10.21769/BioProtoc.2103 PMC8376597

[15] Galon, J. et al. Type, density, and location of immune cells within human colorectal tumors predict clinical outcome. *Science* **313** (5795), 1960–1964. 10.1126/science.1129139 (2006). 17008531 10.1126/science.1129139

[16] Pagès, F. et al. International validation of the consensus immunoscore for the classification of colon cancer: a prognostic and accuracy study. *Lancet* **391** (10135), 2128–2139. 10.1016/S0140-6736(18)30789-X (2018). 29754777 10.1016/S0140-6736(18)30789-X

[17] Auslander, N. et al. Robust prediction of response to immune checkpoint Blockade therapy in metastatic melanoma. *Nat. Med.* **24** (10), 1545–1549. 10.1038/s41591-018-0157-9 (2018). 30127394 10.1038/s41591-018-0157-9 PMC6693632

[18] Shao, Y. et al. Drug co-administration in the tumor immune microenvironment of Hepatocellular carcinoma. *Acupuncture and Herbal Medicine*. 3 (3), 189–199 (2023). 10.1097/HM9.0000000000000074

[19] Yoshihara, K. et al. Inferring tumour purity and stromal and immune cell admixture form expression data. *Nat. Commun.* **4**, 2612. 10.1038/ncomms3612 (2013). 24113773 10.1038/ncomms3612 PMC3826632

[20] Hänzelmann, S., Castelo, R. & Guinney, J. GSVA: gene set variation analysis for microarray and RNA-seq data. *BMC Bioinform.* **14**, 7. 10.1186/1471-2105-14-7 (2013). 10.1186/1471-2105-14-7 PMC3618321 23323831

[21] Chen, D. S. & Mellman, I. Oncology meets immunology: the cancer-immunity cycle. *Immunity* **39** (1), 1–10. 10.1016/j.immuni.2013.07.012 (2013). 23890059 10.1016/j.immuni.2013.07.012

[22] Xu, L. et al. TIP: A web server for resolving tumor immunophenotype profiling. *Cancer Res.* **78** (23), 6575–6580. 10.1158/0008-5472.CAN-18-0689 (2018). 30154154 10.1158/0008-5472.CAN-18-0689

[23] Skrzypczak, M. et al. Modeling oncogenic signaling in colon tumors by multidirectional analyses of microarray data directed for maximization of analytical reliability. *Plos One*. **5** (10), e13091. 10.1371/journal.pone.0013091 (2010). 20957034 10.1371/journal.pone.0013091 PMC2948500

[24] Reumers, J. et al. Gene expression data of patients presenting with concurrent colorectal adenomas and colorectal tumors were obtained using Affymetrix U133 + arrays. *GEO datasets*. (2018).

[25] Mayakonda, A., Lin, D., Assenov, Y., Plass, C. & Koeffler, H. P. Maftools: efficient and comprehensive analysis of somatic variants in cancer. *Genome Res.* **28** (11), 1747–1756. 10.1101/gr.239244.118 (2018). 30341162 10.1101/gr.239244.118 PMC6211645

[26] Mermel, C. H. et al. GISTIC2.0 facilitates sensitive and confident localization of the targets of focal somatic copy-number alteration in human cancers. *Genome Biol*. 12 (4), R41 (2011). 10.1186/gb-2011-12-4-r41 10.1186/gb-2011-12-4-r41 PMC3218867 21527027

[27] Llosa, N. J. et al. The vigorous immune microenvironment of microsatellite instable colon cancer is balanced by multiple counter-inhibitory checkpoints. *Cancer Discov*. **5** (1), 43–51. 10.1158/2159-8290.CD-14-0863 (2015). 25358689 10.1158/2159-8290.CD-14-0863 PMC4293246

[28] Ferkel, S. A. et al. Tumor-infiltrating immune cells in colorectal cancer: immune subtypes, prognostic role and biomarker profiling. *Neoplasia* **59**, 101091. 10.1016/j.neo.2024.101091 (2025). 39642846 10.1016/j.neo.2024.101091 PMC11665540

[29] Mariathasan, S. et al. TGFβ attenuates tumour response to PD-L1 Blockade by contributing to exclusion of T cells. *Nature* **554** (7693), 544–548. 10.1038/nature25501 (2018). 29443960 10.1038/nature25501 PMC6028240

[30] Yu, G., Wang, L., Han, Y. & He, Q. ClusterProfiler: an R package for comparing biological themes among gene clusters. *OMICS* **16** (5), 284–287. 10.1089/omi.2011.0118 (2012). 22455463 10.1089/omi.2011.0118 PMC3339379

[31] Chalmers, Z. R. et al. Analysis of 100,000 human cancer genomes reveals the landscape of tumor mutational burden. *Genome Med.* **9** (1), 34. 10.1186/s13073-017-0424-2 (2017). 28420421 10.1186/s13073-017-0424-2 PMC5395719

[32] Baretti, M. & Le, D. T. DNA mismatch repair in cancer. *Pharmacol. Ther.* **189**, 45–62. 10.1016/j.pharmthera.2018.04.004 (2018). 29669262 10.1016/j.pharmthera.2018.04.004

[33] Ma, S. et al. The interplay between m6A RNA methylation and noncoding RNA in cancer. *J. Hematol. Oncol.* **12** (1), 121. 10.1186/s13045-019-0805-7 (2019). 31757221 10.1186/s13045-019-0805-7 PMC6874823

[34] Niu, X. et al. Landscape of N^6^-Methyladenosine modification patterns in human ameloblastoma. *Front. Oncol.* **10**, 556497. 10.3389/fonc.2020.556497 (2020). 33178585 10.3389/fonc.2020.556497 PMC7592903

[35] Chen, L. et al. Downregulated miR-524-5p participates in the tumor microenvironment of ameloblastoma by targeting the interleukin-33 (IL-33)/suppression of tumorigenicity 2 (ST2) axis. *Med. Sci. Monit.* **26**, e921863. 10.12659/MSM.921863 (2020). 31990904 10.12659/MSM.921863 PMC6998793

[36] Li, J., Liang, L., Yang, Y., Li, X. & Ma, Y. N^6^-methyladenosine as a biological and clinical determinant in colorectal cancer: progression and future direction. *Theranostics* **11** (6), 2581–2593. 10.7150/thno.52366 (2021). 33456561 10.7150/thno.52366 PMC7806471

[37] Nguyen, T. T. T. et al. Tryptophan-dependent and -independent secretions of tryptophanyl - tRNA synthetase mediate innate inflammatory responses. *Cell. Rep.* **42** (1), 111905. 10.1016/j.celrep.2022.111905 (2023). 36640342 10.1016/j.celrep.2022.111905

[38] Jiang, Q. et al. Cell biology of IL-7, a key Lymphotrophin. *Cytokine Growth Factor. Rev.* **16** (4-5), 513–533. 10.1016/j.cytogfr.2005.05.004 (2005). 15996891 10.1016/j.cytogfr.2005.05.004

[39] Fry, T. J. & Mackall, C. L. The many faces of IL-7: from lymphopoiesis to peripheral T cell maintenance. *J. Immunol.* **174** (11), 6571–6576. 10.4049/jimmunol.174.11.6571 (2005). 15905493 10.4049/jimmunol.174.11.6571

[40] Freeden-Jeffry, U. V. et al. Lymphopenia in Interleukin (IL)-7 gene-deleted mice identifies IL-7 as a nonredundant cytokine. *J. Exp. Med.* **181** (4), 1519–1526. 10.1084/jem.181.4.1519 (1995). 7699333 10.1084/jem.181.4.1519 PMC2191954

[41] Peschon, J. J. et al. Early lymphocyte expansion is severely impaired in Interleukin 7 receptor-deficient mice. *J. Exp. Med.* **180** (5), 1955–1960. 10.1084/jem.180.5.1955 (1994). 7964471 10.1084/jem.180.5.1955 PMC2191751

[42] Puel, A., Ziegler, S. F., Buckley, R. H. & Leonard, W. J. Defective IL7R expression in T(-)B(+)NK(+) severe combined immunodeficiency. *Nat. Genet.* **20** (4), 394–397. 10.1038/3877 (1998). 9843216 10.1038/3877

[43] Roifman, C. M., Zhang, J., Chitayat, D. & Sharfe, N. A partial deficiency of interleukin-7R alpha is sufficient to abrogate T-cell development and cause severe combined immunodeficiency. *Blood* **96** (8), 2803–2807. 10.1046/j.1537-2995.2000.40111421.x (2000). 11023514

[44] Thomas, S. J., Snowden, J. A., Zeidler, M. P. & Danson, S. J. The role of JAK/STAT signalling in the pathogenesis, prognosis and treatment of solid tumors. *Br. J. Cancer*. **113** (3), 365–371. 10.1038/bjc.2015.233 (2015). 26151455 10.1038/bjc.2015.233 PMC4522639

[45] Nguyen, T. T. T. et al. Tryptophanyl-tRNA synthetase 1 signals activate TREM-1 via TLR2 and TLR4 in macrophages. *Cell. Rep.* **33** (6), 108367. 10.1016/j.celrep.2020.108367 (2020). 32899943 10.3390/biom10091283 PMC7565148

[46] Lee, H. C. et al. Released tryptophanyl-tRNA synthetase stimulates innate immune responses against viral infection. *J. Virol.* **93** (2), e01291–e01218. 10.1128/JVI.01291-18 (2019). 30355684 10.1128/JVI.01291-18 PMC6321899

[47] Ahn, Y. H. et al. Secreted tryptophanyl-tRNA synthetase as a primary defence system against infection. *Nat. Microbiol.* **2**, 16191. 10.1038/nmicrobiol.2017.15 (2017). 10.1038/nmicrobiol.2016.191 27748732

[48] Zheng, L. et al. Cancer-associated fibroblasts: a pivotal regulator of tumor microenvironment in the context of radiotherapy. *Cell. Commun. Signal.* **23**, 147. 10.1186/s12964-025-02138-7 (2025). 40114180 10.1186/s12964-025-02138-7 PMC11927177

