# Supplementary material for: Progressively altered genes in colorectal carcinogenesis link oncogenesis immune cycle and tumor microenvironment
Source: Sci Rep. 2025 Oct 27;15:37495. doi: 10.1038/s41598-025-21401-y (PMC12559207; doi:10.1038/s41598-025-21401-y)
Supplement: Supplementary file 1 — Supplementary Material 1 [file 41598_2025_21401_MOESM1_ESM.docx]

| Table S1 Wilcoxon rank-sum test results for Normal and Tumor comparisons in TCGA | | | | | |
| --- | --- | --- | --- | --- | --- |
| Gene | Comparison | Pvalue | Effect size (r) | 95% CI lower | 95% CI upper |
| ADAMTSL4 | Normal vs Tumor | <0.0001 | 0.2812 | 1.5831 | 0.9829 |
| F2RL1 | Normal vs Tumor | <0.0001 | 0.3758 | 1.2693 | 0.9575 |
| FCGR1A | Normal vs Tumor | <0.0001 | 0.1943 | 0.7492 | 1.5610 |
| FCGR1CP | Normal vs Tumor | <0.0001 | 0.2239 | 0.9320 | 1.8511 |
| HECW2 | Normal vs Tumor | <0.0001 | 0.3084 | 1.0269 | 1.5573 |
| IFITM3 | Normal vs Tumor | <0.0001 | 0.3565 | 1.5164 | 2.0296 |
| OPLAH | Normal vs Tumor | 0.0013 | 0.1218 | 0.1395 | 0.5526 |
| SECTM1 | Normal vs Tumor | <0.0001 | 0.4074 | 3.3651 | 2.6133 |
| SERPINA1 | Normal vs Tumor | 0.9934 | 0.0003 | 0.3950 | 0.3955 |
| SLC16A3 | Normal vs Tumor | 0.0002 | 0.1385 | 0.1940 | 0.6376 |
| WARS1 | Normal vs Tumor | <0.0001 | 0.2147 | 0.4326 | 0.8277 |

| Table S2 Wilcoxon rank-sum test results for clinical information comparisons in TCGA | | | | | |
| --- | --- | --- | --- | --- | --- |
| Gene | Comparison | Pvalue | Effect size (r) | 95% CI lower | 95% CI upper |
| WARS1 | Stage I vs II | 0.2895 | 0.0608 | 0.1012 | 0.3012 |
| WARS1 | Stage I vs III | 0.0508 | 0.1261 | 0.0009 | 0.4380 |
| WARS1 | Stage I vs IV | <0.0001 | 0.3133 | 0.2689 | 0.7191 |
| WARS1 | Stage II vs III | 0.2123 | 0.0660 | 0.0640 | 0.2943 |
| WARS1 | Stage II vs IV | <0.0001 | 0.2286 | 0.2028 | 0.5854 |
| WARS1 | Stage III vs IV | 0.0052 | 0.1829 | 0.0836 | 0.4698 |
| WARS1 | M0 vs M1 | <0.0001 | 0.1794 | 0.2005 | 0.5475 |
| SERPINA1 | Stage I vs II | 0.0528 | 0.1112 | 0.4293 | 0.0052 |
| SERPINA1 | Stage I vs III | 0.0017 | 0.2025 | 0.8277 | 0.3122 |
| SERPINA1 | Stage I vs IV | <0.0001 | 0.2980 | 1.0592 | 0.5855 |
| SERPINA1 | Stage II vs III | 0.0485 | 0.1044 | 0.3824 | 0.0013 |
| SERPINA1 | Stage II vs IV | 0.0013 | 0.1872 | 0.6301 | 0.2594 |
| SERPINA1 | Stage III vs IV | 0.2903 | 0.0693 | 0.2558 | 0.1955 |
| SECTM1 | Stage I vs II | 0.2078 | 0.0724 | 0.6832 | 0.1458 |
| SECTM1 | Stage I vs III | 0.6559 | 0.0288 | 0.2832 | 0.4586 |
| SECTM1 | Stage I vs IV | 0.1448 | 0.1090 | 0.1068 | 0.7285 |
| SECTM1 | Stage II vs III | 0.0487 | 0.1043 | 0.0022 | 0.6932 |
| SECTM1 | Stage II vs IV | 0.0070 | 0.1567 | 0.1674 | 0.9851 |
| SECTM1 | Stage III vs IV | 0.1981 | 0.0843 | 0.1333 | 0.6027 |
| FCGR1CP | Stage I vs II | 0.0001 | 0.2223 | 1.7085 | 0.3716 |
| FCGR1CP | Stage I vs III | 0.0157 | 0.1559 | 1.3581 | -<0.0001 |
| FCGR1CP | Stage I vs IV | 0.0365 | 0.1563 | 1.4074 | -<0.0001 |
| FCGR1CP | Stage II vs III | 0.0741 | 0.0946 | -<0.0001 | 0.7109 |
| FCGR1CP | Stage II vs IV | 0.1512 | 0.0836 | 0.0535 | 0.8137 |
| FCGR1CP | Stage III vs IV | 0.9084 | 0.0075 | 0.4479 | 0.4851 |
| FCGR1A | Stage I vs II | 0.0090 | 0.1500 | 0.8913 | 0.1300 |
| FCGR1A | Stage I vs III | 0.0972 | 0.1071 | 0.7383 | 0.0721 |
| FCGR1A | Stage I vs IV | 0.5754 | 0.0419 | 0.6011 | 0.3307 |
| FCGR1A | Stage II vs III | 0.2916 | 0.0558 | 0.1507 | 0.5039 |
| FCGR1A | Stage II vs IV | 0.0483 | 0.1148 | 0.0029 | 0.7440 |
| FCGR1A | Stage III vs IV | 0.3029 | 0.0675 | 0.2041 | 0.6311 |

| Table S3 Wilcoxon rank-sum test results for Nromal, Adenoma and Cancer comparisons in GEO | | | | | | |
| --- | --- | --- | --- | --- | --- | --- |
| Gene | Comparison | Pvalue | Effect size (r) | 95% CI lower | 95% CI upper | Dataset |
| HECW2 | Adenoma vs Cancer | <0.0001 | 0.7083 | 0.4912 | 0.2610 | GSE20916 |
| SERPINA1 | Adenoma vs Cancer | <0.0001 | 0.6981 | 0.2121 | 0.3015 |  |
| SECTM1 | Adenoma vs Cancer | <0.0001 | 0.5863 | 0.4789 | 0.2718 |  |
| WARS1 | Adenoma vs Cancer | <0.0001 | 0.5373 | 0.2097 | 0.1222 |  |
| F2RL1 | Adenoma vs Cancer | <0.0001 | 0.4717 | 0.1207 | 0.3276 |  |
| IFITM3 | Adenoma vs Cancer | <0.0001 | 0.4552 | 0.1034 | 0.0490 |  |
| FCGR1A | Adenoma vs Cancer | <0.0001 | 0.3754 | 0.3555 | 0.0659 |  |
| SLC16A3 | Adenoma vs Cancer | 0.0002 | 0.3492 | 0.2517 | 0.0814 |  |
| OPLAH | Adenoma vs Cancer | 0.1095 | 0.1519 | 0.2286 | 0.0244 |  |
| SECTM1 | Normal vs Adenoma | <0.0001 | 0.8071 | 0.4914 | 0.6468 |  |
| SERPINA1 | Normal vs Adenoma | <0.0001 | 0.7625 | 0.3891 | 0.2603 |  |
| IFITM3 | Normal vs Adenoma | <0.0001 | 0.7091 | 0.1811 | 0.1246 |  |
| SLC16A3 | Normal vs Adenoma | 0.0060 | 0.3091 | 0.0452 | 0.2588 |  |
| WARS1 | Normal vs Adenoma | 0.0117 | 0.2835 | 0.0958 | 0.0158 |  |
| FCGR1A | Normal vs Adenoma | 0.0246 | 0.2529 | 0.0040 | 0.3127 |  |
| OPLAH | Normal vs Adenoma | 0.0419 | 0.2289 | 0.0040 | 0.2495 |  |
| F2RL1 | Normal vs Adenoma | 0.0634 | 0.2089 | 0.0093 | 0.1468 |  |
| HECW2 | Normal vs Adenoma | 0.4197 | 0.0908 | 0.0083 | 0.0764 |  |
| SERPINA1 | Adenoma vs Cancer | <0.0001 | 0.3836 | 0.1155 | 0.2593 | GSE117606 |
| F2RL1 | Adenoma vs Cancer | 0.0009 | 0.2782 | 0.0352 | 0.1184 |  |
| WARS1 | Adenoma vs Cancer | 0.0024 | 0.2542 | 0.0948 | 0.0177 |  |
| OPLAH | Adenoma vs Cancer | 0.0154 | 0.2025 | 0.0147 | 0.1255 |  |
| ADAMTSL4 | Adenoma vs Cancer | 0.0813 | 0.1458 | 0.0914 | 0.0051 |  |
| HECW2 | Adenoma vs Cancer | 0.1128 | 0.1326 | 0.1058 | 0.0102 |  |
| SLC16A3 | Adenoma vs Cancer | 0.1604 | 0.1174 | 0.0753 | 0.0117 |  |
| SECTM1 | Adenoma vs Cancer | 0.7116 | 0.0309 | 0.0915 | 0.0618 |  |
| SERPINA1 | Normal vs Adenoma | <0.0001 | 0.6411 | 0.3552 | 0.2292 |  |
| SECTM1 | Normal vs Adenoma | <0.0001 | 0.5223 | 0.1857 | 0.3349 |  |
| ADAMTSL4 | Normal vs Adenoma | <0.0001 | 0.4692 | 0.1049 | 0.2132 |  |
| F2RL1 | Normal vs Adenoma | <0.0001 | 0.3388 | 0.0426 | 0.1205 |  |
| SLC16A3 | Normal vs Adenoma | 0.0004 | 0.3046 | 0.0886 | 0.0269 |  |
| OPLAH | Normal vs Adenoma | 0.0015 | 0.2746 | 0.1283 | 0.0327 |  |
| WARS1 | Normal vs Adenoma | 0.0406 | 0.1769 | 0.0653 | 0.0012 |  |
| HECW2 | Normal vs Adenoma | 0.2619 | 0.0969 | 0.1113 | 0.0263 |  |

| Table S4 Wilcoxon rank-sum test results for WARS1 in Normal, Adenoma and Cancer | | | | |
| --- | --- | --- | --- | --- |
| Comparison | Pvalue | Effect size (r) | 95% CI lower | 95% CI upper |
| Normal vs Adenoma | 0.04573124 | 0.342631112 | 0.001090694 | Inf |
| Adenoma vs Cancer | 0.04557358 | 0.230861759 | 0.000610453 | Inf |

| Table S5 Wilcoxon rank-sum test results for tumor microenvironment | | | | | |
| --- | --- | --- | --- | --- | --- |
| Variable | Comparison | Pvalue | Effect size (r) | 95% CI lower | 95% CI upper |
| StromalScore | Hihg vs Low | <0.0001 | 0.3651 | 407.4314 | 622.7739 |
| ImmuneScore | Hihg vs Low | <0.0001 | 0.5075 | 548.4686 | 729.8975 |
| TumorPurity | Hihg vs Low | <0.0001 | 0.4647 | 0.1096 | 0.0792 |

| Table S6 Wilcoxon rank-sum test results for immune cells | | | | | |
| --- | --- | --- | --- | --- | --- |
| Immune cell | Comparison | Pvalue | Effect size (r) | 95% CI lower | 95% CI upper |
| Natural killer T cell | High vs Low | <0.0001 | 0.5380 | 0.0570 | 0.0743 |
| Activated CD4 T cell | High vs Low | <0.0001 | 0.5361 | 0.0561 | 0.0733 |
| Effector memeory CD8 T cell | High vs Low | <0.0001 | 0.5291 | 0.0635 | 0.0829 |
| Activated CD8 T cell | High vs Low | <0.0001 | 0.5235 | 0.0547 | 0.0725 |
| T follicular helper cell | High vs Low | <0.0001 | 0.5207 | 0.0511 | 0.0672 |
| Myeloid derived suppressor cell | High vs Low | <0.0001 | 0.5193 | 0.0712 | 0.0944 |
| Type 1 T helper cell | High vs Low | <0.0001 | 0.4876 | 0.0427 | 0.0580 |
| Activated dendritic cell | High vs Low | <0.0001 | 0.4827 | 0.0612 | 0.0822 |
| Type 2 T helper cell | High vs Low | <0.0001 | 0.4758 | 0.0403 | 0.0541 |
| Central memory CD4 T cell | High vs Low | <0.0001 | 0.4752 | 0.0197 | 0.0273 |
| Gamma delta T cell | High vs Low | <0.0001 | 0.4671 | 0.0327 | 0.0450 |
| Central memory CD8 T cell | High vs Low | <0.0001 | 0.4655 | 0.0380 | 0.0520 |
| Immature B cell | High vs Low | <0.0001 | 0.4607 | 0.0961 | 0.1331 |
| Regulatory T cell | High vs Low | <0.0001 | 0.4580 | 0.0622 | 0.0867 |
| Macrophage | High vs Low | <0.0001 | 0.4495 | 0.0589 | 0.0823 |
| Natural killer cell | High vs Low | <0.0001 | 0.4436 | 0.0319 | 0.0446 |
| Immature dendritic cell | High vs Low | <0.0001 | 0.3875 | 0.0232 | 0.0343 |
| Effector memeory CD4 T cell | High vs Low | <0.0001 | 0.3802 | 0.0349 | 0.0525 |
| Activated B cell | High vs Low | <0.0001 | 0.3788 | 0.0869 | 0.1326 |
| Mast cell | High vs Low | <0.0001 | 0.3605 | 0.0599 | 0.0910 |
| Neutrophil | High vs Low | <0.0001 | 0.3590 | 0.0651 | 0.0996 |
| Plasmacytoid dendritic cell | High vs Low | <0.0001 | 0.2824 | 0.0130 | 0.0229 |
| CD56dim natural killer cell | High vs Low | <0.0001 | 0.2608 | 0.0097 | 0.0181 |
| Monocyte | High vs Low | <0.0001 | 0.2143 | 0.0071 | 0.0153 |
| Type 17 T helper cell | High vs Low | <0.0001 | 0.2134 | 0.0137 | 0.0299 |
| Eosinophil | High vs Low | <0.0001 | 0.2061 | 0.0158 | 0.0356 |
| CD56bright natural killer cell | High vs Low | <0.0001 | 0.1903 | 0.0041 | 0.0101 |
| Memory B cell | High vs Low | <0.0001 | 0.1621 | 0.0102 | 0.0304 |

| Table S7 Wilcoxon rank-sum test results for cancer immunity cycle | | | | | |
| --- | --- | --- | --- | --- | --- |
| Pathway | Comparison | Pvalue | Effect size (r) | 95% CI lower | 95% CI upper |
| Killing of cancer cells | High vs Low | <0.0001 | 0.6042 | 0.4432 | 0.5531 |
| Recognition of cancer cells by T cells | High vs Low | <0.0001 | 0.6027 | 0.5089 | 0.6324 |
| NK cell recruiting | High vs Low | <0.0001 | 0.5762 | 0.6054 | 0.7820 |
| T cell recruiting | High vs Low | <0.0001 | 0.5548 | 0.5589 | 0.7313 |
| Macrophage recruiting | High vs Low | <0.0001 | 0.5531 | 0.6194 | 0.8320 |
| CD8 Tcell recruiting | High vs Low | <0.0001 | 0.5527 | 0.4969 | 0.6411 |
| Infiltration of Immune cells into tumors | High vs Low | <0.0001 | 0.5506 | 0.4004 | 0.5130 |
| Dendritic cell recruiting | High vs Low | <0.0001 | 0.5442 | 0.5826 | 0.7690 |
| Priming and activation | High vs Low | <0.0001 | 0.5400 | 0.4528 | 0.6108 |
| Cancer antigen presentation | High vs Low | <0.0001 | 0.5014 | 0.2909 | 0.3877 |
| Th1 cell recruiting | High vs Low | <0.0001 | 0.4974 | 0.5407 | 0.7325 |
| Release of cancer cell antigens | High vs Low | <0.0001 | 0.4825 | 0.2301 | 0.3137 |
| Monocyte recruiting | High vs Low | <0.0001 | 0.4570 | 0.4623 | 0.6538 |
| CD4 Tcell recruiting | High vs Low | <0.0001 | 0.4206 | 0.3968 | 0.5691 |
| B cell recruiting | High vs Low | <0.0001 | 0.3912 | 0.3138 | 0.5468 |
| Th2 cell recruiting | High vs Low | <0.0001 | 0.3277 | 0.2880 | 0.4803 |
| Treg cell recruiting | High vs Low | <0.0001 | 0.3045 | 0.2276 | 0.4019 |
| Th17 cell recruiting | High vs Low | <0.0001 | 0.2615 | 0.1749 | 0.3219 |
| MDSC recruiting | High vs Low | <0.0001 | 0.2143 | 0.1257 | 0.3135 |
| Neutrophil recruiting | High vs Low | <0.0001 | 0.1807 | 0.1016 | 0.2702 |
| Eosinophil recruiting | High vs Low | 0.0013 | 0.1282 | 0.0503 | 0.2156 |
| Th22 cell recruiting | High vs Low | 0.7159 | 0.0145 | 0.0659 | 0.1048 |
| Basophil recruiting | High vs Low | 0.8910 | 0.0055 | 0.0817 | 0.0718 |

| Table S8 Wilcoxon rank-sum test results for known biological signatures | | | | | |
| --- | --- | --- | --- | --- | --- |
| Pathway | Comparison | Pvalue | Effect size (r) | 95% CI lower | 95% CI upper |
| Immune checkpoint | High vs Low | <0.0001 | 0.6201 | 0.7785 | 0.9759 |
| CD8 T effector | High vs Low | <0.0001 | 0.5786 | 0.6353 | 0.8291 |
| Antigen processing machinery | High vs Low | <0.0001 | 0.4775 | 0.5078 | 0.7516 |
| Angiogenesis | High vs Low | <0.0001 | 0.2985 | 0.2591 | 0.4754 |
| Cell cycle regulators | High vs Low | <0.0001 | 0.2978 | 0.1643 | 0.2774 |
| EMT2 | High vs Low | <0.0001 | 0.2978 | 0.2262 | 0.4088 |
| EMT3 | High vs Low | <0.0001 | 0.2963 | 0.2027 | 0.3438 |
| Cell cycle | High vs Low | <0.0001 | 0.2771 | 0.1361 | 0.2476 |
| Pan-F-TBRs | High vs Low | <0.0001 | 0.2757 | 0.1628 | 0.3041 |
| DNA replication | High vs Low | <0.0001 | 0.2211 | 0.1199 | 0.2676 |
| Nucleotide excision repair | High vs Low | <0.0001 | 0.2144 | 0.0991 | 0.2160 |
| Mismatch repair | High vs Low | <0.0001 | 0.2124 | 0.1123 | 0.2502 |
| EMT1 | High vs Low | <0.0001 | 0.2109 | 0.1132 | 0.2450 |
| Fanconi anemia | High vs Low | <0.0001 | 0.1800 | 0.0751 | 0.1928 |
| DNA damage repair | High vs Low | <0.0001 | 0.1720 | 0.0621 | 0.1693 |
| Homologous recombination | High vs Low | <0.0001 | 0.1652 | 0.0698 | 0.1944 |
| KEGG discovered histones | High vs Low | 0.0184 | 0.0940 | 0.0866 | 0.0079 |
| FGFR3-related genes | High vs Low | 0.0628 | 0.0743 | 0.1338 | 0.0032 |
| WNT target | High vs Low | 0.0749 | 0.0711 | 0.0066 | 0.1352 |
